# Computational modeling of AMPK and mTOR crosstalk in glutamatergic synapse calcium signaling

**DOI:** 10.1038/s41540-023-00295-4

**Published:** 2023-07-17

**Authors:** A. Leung, P. Rangamani

**Affiliations:** 1 Chemical Engineering Graduate Program, University of California San Diego, La Jolla, CA USA; 2 Department of Mechanical and Aerospace Engineering, University of California San Diego, La Jolla, CA USA

**Keywords:** Time series, Computer modelling

## Abstract

Neuronal energy consumption is vital for information processing and memory formation in synapses. The brain consists of just 2% of the human body’s mass, but consumes almost 20% of the body’s energy budget. Most of this energy is attributed to active transport in ion signaling, with calcium being the canonical second messenger of synaptic transmission. Here, we develop a computational model of synaptic signaling resulting in the activation of two protein kinases critical in metabolic regulation and cell fate, AMP-Activated protein kinase (AMPK) and mammalian target of rapamycin (mTOR) and investigate the effect of glutamate stimulus frequency on their dynamics. Our model predicts that frequencies of glutamate stimulus over 10 Hz perturb AMPK and mTOR oscillations at higher magnitudes by up to 36% and change the area under curve (AUC) by 5%. This dynamic difference in AMPK and mTOR activation trajectories potentially differentiates high frequency stimulus bursts from basal neuronal signaling leading to a downstream change in synaptic plasticity. Further, we also investigate the crosstalk between insulin receptor and calcium signaling on AMPK and mTOR activation and predict that the pathways demonstrate multistability dependent on strength of insulin signaling and metabolic consumption rate. Our predictions have implications for improving our understanding of neuronal metabolism, synaptic pruning, and synaptic plasticity.

## Introduction

**Fig. 1:**
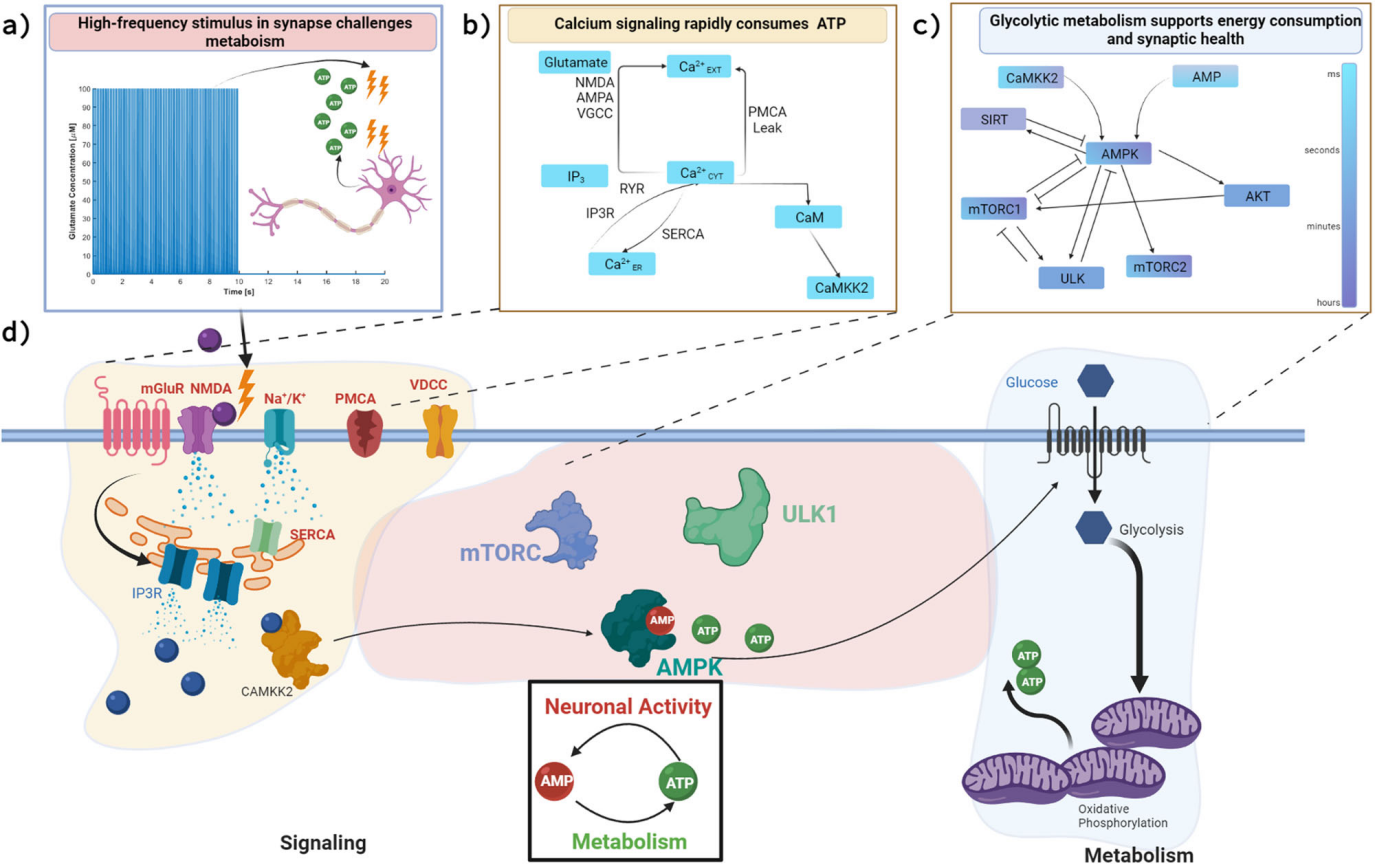
Synaptic signaling consumes energy to transduce neuronal signals and support neuronal function. During high-frequency synaptic signaling, represented with glutamate stimulus in (**a**), proportionally larger quantities of ATP are allocated to restoring resting potential of ions, like those shown for calcium illustrated in (**b**). At high frequencies, this may challenge the energy production capacity of neurons, which utilize glycolysis and oxidative phosphorylation to convert glucose to pyruvate and then produce ATP in dendritic mitochondria. The decreasing cellular energy state (higher AMP/ATP ratio) promotes the phosphorylation of AMPK, a kinase which promotes the production of cellular ATP, but also has an intricate feedback loop with mTORC1 and mTORC2 downstream of the insulin signaling cascade, shown in (**c**), which have implications in protein translation and synaptic plasticity. In this work, we develop and analyze a computational model to study the interactions of these pathways, illustrated in (**d**), in response to a synaptic stimulus across several timescales. Created with BioRender.com.

Calcium signal transduction in dendritic spines during neuronal signaling is closely linked to synaptic plasticity^1–5^. Synaptic plasticity is the structural and molecular modification of synapses resulting in sustained changes in synaptic signaling strength^2,6,7^. There are many potential mechanisms by which calcium signaling leads to long-term plasticity (LTP), the process by which synaptic connections are strengthened with high-frequency stimulus, but the relative contribution of these different mechanisms is unclear^8^. One such mechanism is metabolic plasticity, which is the adaptation of cellular energy production in response to metabolic stress associated with calcium signaling^9–11^ and is the focus of our current work. Electrical signaling, including the reversal of presynaptic and postsynaptic ion fluxes, accounts for a vast majority of ATP consumption in mammalian neuron signaling^12,13^. During an action potential, energy consumption is estimated to transiently increase up to 130 percent over basal ATP flux with most of this energy attributed to ion signaling, actin and microtubule turnover, and lipid and protein translation^12–14^. Therefore, the coupling of metabolic plasticity with ion dynamics is necessary for dendritic spines due to a drastic increase in energy consumption during neuronal signaling^11^. In addition, there are a variety of energetically expensive processes in the dendritic spine that are necessary to induce long-term potentiation (LTP) including actin remodeling, protein translation, endocytosis, and exocytosis^14^. The frequency of neurotransmitter signaling (Fig. 1a) is believed to be critical in the induction of LTP in dendritic spines and also increases the consumption of cellular energy due to the increased rate of active transport of ions to restore resting potentials^13^.

The two primary means of energy production within neurons are glycolysis and oxidative phosphorylation in the mitochondria^15,16^. Glycolysis, the metabolism of glucose in the cytosol, is often upregulated during neuronal stimulus through increased import of glucose from extracellular space and protein kinase-dependent activation of enzymes.^17,18^. Mitochondrial oxidative phosphorylation produces a large portion of cellular energy that supports increased energy demand. However, oxidative phosphorylation requires pyruvate and oxygen consumption to generate ATP in the mitochondrial matrix through mitochondrial metabolism of pyruvate driving the electron transport chain. In actively signaling neurons, mitochondria are observed to form spatially stable pools near the base of dendritic spine, suggesting that they can cater to the increased ATP demand by localizing ATP production proximal to the dendritic spine^19^. It has also been observed that calcium influx from the extracellular space impacts the mitochondrial membrane potential, resulting in higher ATP generation^20^. This provides a route for calcium, the main second messenger system in signaling, to interact directly with the energy production in a frequency-dependent manner. The thermodynamic constraints of ATP production in the mitochondria are explored in Garcia et al. ^21^.

In actively signaling regions of neurons, mitochondria often form spatially stable pools proximal to the axonal bouton and the dendritic spine^19^. While axons have a generally higher density of mitochondria due to their need to support high rates of neurotransmitter exocytosis, dendrites during signaling are able to stabilize mitochondria to support high rates of energy consumption^19,22^. Mitochondrial motility and distribution are critical for supporting synaptic plasticity by supplying energy at synapses^23^. Computational modeling has demonstrated that key morphological features, along with signaling characteristics such as frequency, duration, and calcium amplitude, may enhance mitochondrial plasticity^24^.

Calcium influx (Fig. 1b) indirectly modulates neuronal glycolysis and mitochondrial metabolism via enzymatic activation (CAMKII^25^), but the means by which neurons regulate the energy production local to the dendritic spine remains unexplored^20^. A possible avenue for feedback between neuronal energy consumption and metabolic production is through cellular energy sensors and kinases. Adenine monophosphate-activated Protein Kinase (AMPK) is a candidate molecule for the coupling between calcium dynamics and cellular metabolism due to its unique function as the cellular energy stress sensor^20,26^. The *γ* subunit of this heterotrimeric protein is able to adapt to changing ratios of AMP and ATP, exposing a phosphorylation site and enabling kinase functionality^27^. Upstream protein kinases of AMPK include CAMKK2, LKB1, AKT, mTOR, and PI3K, and may regulate AMPK activity depending on phosphorylation site^25,28,29^. The downstream targets of AMPK include phosphofructokinase (PFK), mitochondrial biogenesis, and inhibition of energy-consuming pathways like lipid synthesis and gluconeogenesis. Calcium-Calmodulin activated Kinase Kinase 2 (CaMKK2) enables direct feedback from the calcium signaling pathway to AMPK. Calcium also creates an energy imbalance because of increased energy demand by ion pumps and exchangers, which increases AMP/ATP ratio and activates AMPK^11,25,26,30^. AMPK activated by calcium, phosphonucleotide binding, or allosteric activation pathways leads to feedback to dynamically enhance energy production. AMPK has been shown to increase the import of glucose into neurons by promoting trafficking of glucose import receptors GLUT3 to the plasma membrane^31^. Furthermore, AMPK upregulates glycolysis by modulating PFK activity during energy stress in cardiac cells as well as mitochondrial morphology and motility in neuronal axons^32,33^.

One critical downstream target of AMPK is mammalian target of rapamycin (mTOR); select interactions are shown in Fig. 1c. mTOR is a well-conserved protein across mammalian cell types, but occupies a unique niche in neurons. While classically mTOR forms a protein complex that regulates protein translation and mitophagy^34^, mTOR has also been shown to be critical in synapse formation^11,34–36^. In presynaptic boutons, mTOR forms a complex with Rictor, mTORC2, which is necessary for bouton formation^34^. In postsynaptic dendritic spines, mTOR forms a complex with Raptor, mTORC1^11^. mTORC1 has been shown to influence the translation and transport of *α*-Amino-3-hydroxy-5-methyl-4-isoxazolepropionic acid (AMPA) receptors and scaffolding proteins needed for the clustering of AMPAR, a critical step in synaptic plasticity^37^. Abolishment of mTOR activity in both presynaptic and postsynaptic cells has been performed in live neurons and shows a decrease in the number of spines and plastic ability^35,37^. The exact mechanisms by which the calcium-AMPK-mTOR signaling axis is regulated in neurons are yet to be explored. In particular, exploration is needed to bridge the activity of calcium signaling, which typically occurs on the timescale of milliseconds, to the function of protein kinases (AMPK, mTOR, AKT), which can occur on the timescale of hours to days. AMPK and mTOR are also both downstream targets of the insulin receptor substrate (IRS) signaling cascade, which can bridge the intracellular response to a systems level change in insulin. Downstream of mTORC1 are several transcription regulators, for example, Sirtuin1 (SIRT1) has been shown to regulate neuroprotective pathways and may have a role in dendritic spine formation^38,39^. A multi-timescale model that represents these different systems can shed light into the crosstalk between these two pathways.

In this work, we use computational modeling to explore how the calcium-AMPK-mTOR signaling axis could couple neuronal energy states and mTOR activity. Computational modeling has vastly contributed to our understanding of neuronal signaling and synaptic plasticity^1–3,40–43^. However, very few of these models feature the importance of metabolic feedback mechanisms known to be critical in synaptic formation and activity. We built our model based on prior models in the literature, utilizing a calcium signaling model that incorporates the effect of endoplasmic reticulum (ER) and mitochondria on calcium signaling and mitochondrial ATP production^44^. We complement this calcium model with a model linking cellular energy state via adenine nucleotide balance with AMPK activation^45^. Finally, we explore the AMPK and mTOR crosstalk, by integrating the insulin signaling model within a calcium and metabolism model^29^.

Our model predicts that a wide range of stability behavior is possible depending on glutamate stimulus, neuronal energy consumption, and the behavior of downstream components of the insulin signaling pathway. Glutamate signaling frequency can influence key characteristics in the dynamics of vital protein kinases and can drastically increase the magnitude of AMPK and mTOR phosphorylation and total signaling observed during stimulus. In addition, we observe that internal metabolism (ATP hydrolysis), external metabolism (insulin signaling), and neuronal frequency directly influence the system response, with parameter-dependent oscillations. The multiple stable states of this system implies that the crosstalk between extracellular signals (Fig. 1d), such as glutamate and insulin, culminate in changes to internal metabolic systems such as AMPK and mTOR activation rates. Further exploration of the crosstalk between pathways may elucidate the relation between neuronal metabolism and signal processing in dendrites.

## Results

Within a signaling synapse, the presynaptic neuron releases glutamate vesicles from low frequencies (0.1 to 1 Hz) to high frequencies (10 to 100 Hz). Low-frequency signaling is often associated with long-term depression and synaptic pruning, while high-frequency signaling is believed to induce long-term potentiation (LTP), the mechanism behind neuronal learning^46,47^. Each pulse of glutamate triggers a calcium influx into the post synaptic site requiring a significant cellular energy cost in the form of ATP consumption for the restoration of resting ion potentials and various housekeeping processes (Fig. 1b). As frequencies rise, there may be a critical point at which the ATP production from neuronal metabolism is overwhelmed by the energy demand. At this critical juncture, if the neuron is unable to adequately scale energy production, we hypothesize that the neuron may not be able to form sustained LTP and opt to undergo LTD. The metabolic plasticity, or the ability of the neuron to scale metabolic production to energetic demand, must be modeled alongside the closely coupled calcium signaling cascade to understand how the neuron is able to induce synaptic plasticity during periods of extreme energy stress. In what follows, we investigate the crosstalk between synaptic signaling and metabolic plasticity.

### Model constraints and parametric sensitivity analysis

We first analyzed the system behavior in the absence of any glutamate stimulus to investigate how the coupled signaling networks behave. After a brief initialization period, we see a rapid equilibration of all species to an apparent steady state, shown in Supplementary Figure 1. Many trajectories on the short, millisecond to second, timescale equilibrate nearly instantaneously including calcium, receptors, AMP/ATP, and AMPK. However, several species, like mTORC1, mTORC2, and IP3, take much longer to reach a steady state, on the second to minute timescale. Since the initial conditions are misaligned with the steady state determined by key parameters, there is a brief initialization period before equilibration. For example, as the AMP/ATP ratio is much higher than the steady-state value, this induces AMPK activity which leads to mTOR and AKT activity. Nevertheless, the resulting transient behavior fades after 200 seconds as the dampened oscillations reduce to a fixed point, representing the system at rest. All subsequent simulations use this resting state as initial conditions before stimulus.

**Fig. 2:**
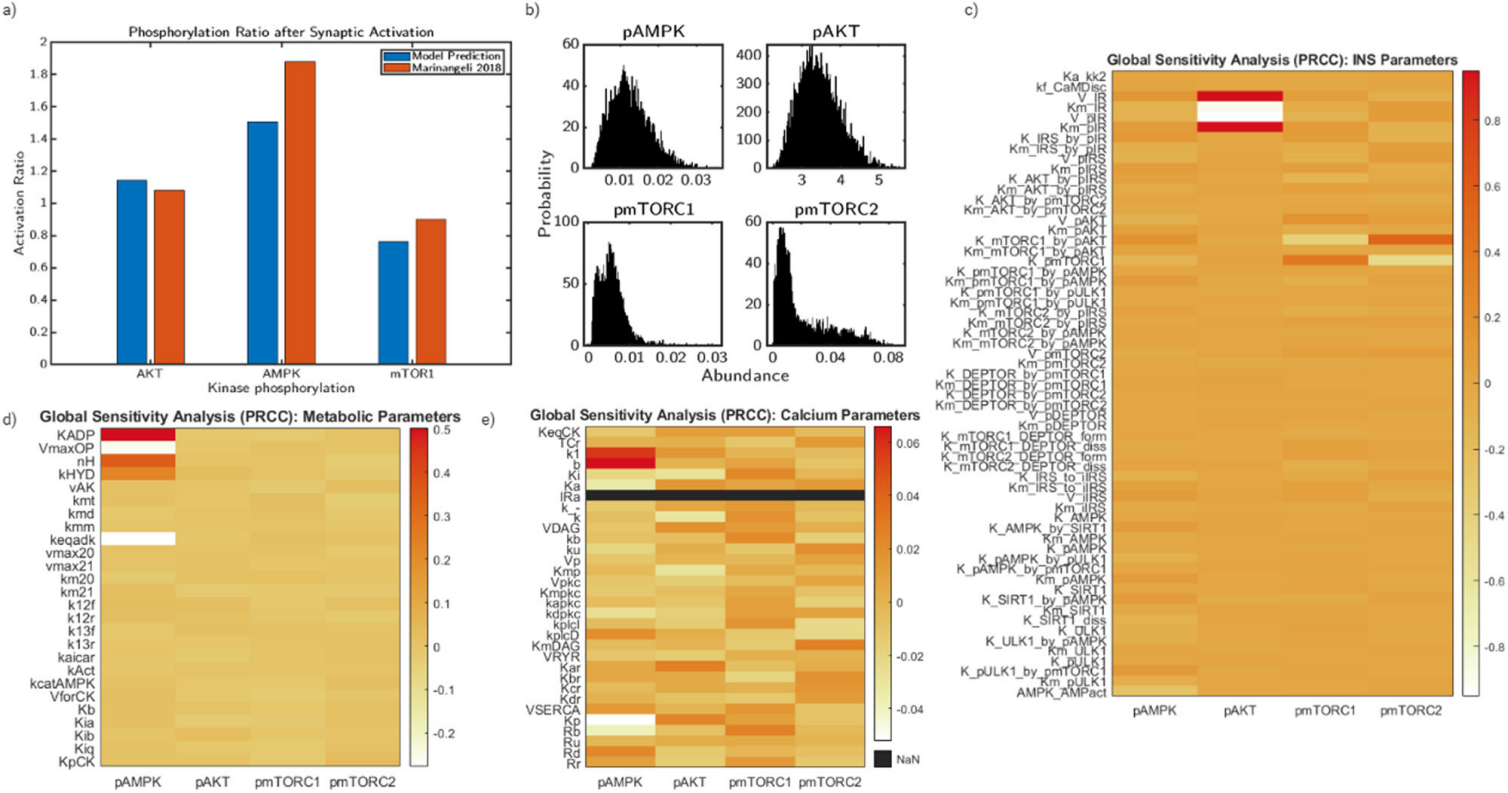
Sensitivity analysis reveal key parameters regulating system behavior. The model describing AMPK and mTOR phosphorylation due to glutamate-stimulated calcium influx contains 163 parameters and 60 equations in a well-mixed model. **a** Comparisons between model predictions of phosphorylation ratios relative to initial state after 10 Hz synaptic activation and experimental results from Marinangeli et al. 2018 primary neuron cells stimulated via Biciculin/4AP protocol. **b** Probability density functions of steady-state concentrations of pAMPK, pAKT, pmTORC1, and pmTORC2 resulting from a global sensitivity analysis of 10,000 parameter values for each 163 parameters in a 20% range. **c** Heatmap of PRCC values describing the correlation between parameter value and system output for AMPK, AKT, mTORC1, and mTORC2 phosphorylation for a subset of parameters belonging to the insulin signaling system. **d** Heatmap of PRCC values describing the correlation between parameter value and system output for AMPK, AKT, mTORC1, and mTORC2 phosphorylation for a subset of parameters belonging to the neuronal metabolism system. **e** Heatmap of PRCC values describing the correlation between parameter value and system output for AMPK, AKT, mTORC1, and mTORC2 phosphorylation for a subset of parameters belonging to the calcium signaling system. Color scale represents PRCC values.

The steady-state behavior of the model is dependent on both parameters and initial conditions. However, due to the complexity of the pathway and the large amount of species and parameters, not all parameters have an equivalent effect on the end result of the model. To understand the complexity of the biological system posed in Fig. 1 we first attempted to reproduce findings of experimental works. In Fig. 2a, we compared the simulation of AMPK, mTORC1, and AKT phosphorylation relative to steady state to the experimental results of Marinangeli et al. ^26^. Our model predictions under 10 Hz of glutamate stimulus align well with their experimental values of differentiated primary neurons stimulated with Bic/4-AP protocol. However, to characterize the uncertainty of the model predictions, we then quantified the effect of parameters on model output.

Through global sensitivity analysis of the system using the PRCC method, we obtained correlation values of each parameter to the model predictions of AMPK, AKT, mTORC1, and mTORC2 phosphorylation. In Fig. 2b, we show histograms of the steady-state values of the model predictions as a probability density function in which the x-axis denotes the steady state concentration and the y-axis denotes the number of parameter sets that predict the steady state. For AMPK concentration, the predictions are well distributed with a mean value of 0.011 μM. AKT also showed similar characteristics, with a mean value of 4.5 nM. This is consistent with expected model behavior as AKT is directly downstream of AMPK. mTORC1 and mTORC2 are both skewed heavily left with mean values of 0.012 and 0.07 μM, respectively. In Fig. 2c–e, we plot heatmaps for the PRCC values of each parameter in the model and the effect on steady-state values of AMPK, AKT, mTORC1, and mTORC2 phosphorylation. We grouped the parameters into sets corresponding to their most relevant biological pathway. Figure 2c corresponds to the parameters most closely related to insulin receptor signaling, originally derived in Sadria et al. ^29^. While most values are close to the mean value, parameters downstream of AKT appear to have the most influence on mTORC1 and mTORC2. AKT is most strongly impacted by V_*IR*_, a parameter which represents the intercellular activity of the insulin receptor signaling and will be investigated.

Next, in Fig. 2d, we plot the PRCC heatmap for the parameters associated with metabolism. For this segment of parameters, AMPK is most strongly impacted by oxidative phosphorylation parameters and the AMPK activation parameter. AKT, mTORC1, and mTORC2 are not strongly influenced by metabolic parameters at steady state. Finally, in Fig. 2e, we plot the PRCC heatmap for calcium-related parameter values. The magnitude of these terms are very small relative to the effect shown in Fig. 2c, d. Overall, we found that the model is very sensitive to parameters corresponding to oxidative phosphorylation, ATP consumption, and insulin signaling. While this implies that the model output is not particularly sensitive to calcium values at steady state, this sensitivity analysis uses simulations without stimulus to obtain steady state. The importance of many calcium parameters is captured by glutamatergic stimulus, which is explored in the following sections. Furthermore, we then take the results of the sensitivity analysis and inform further analysis by selecting three parameters of key biological importance.

### Low-frequency neuronal stimulus reveals low amplitude transient behavior

**Fig. 3:**
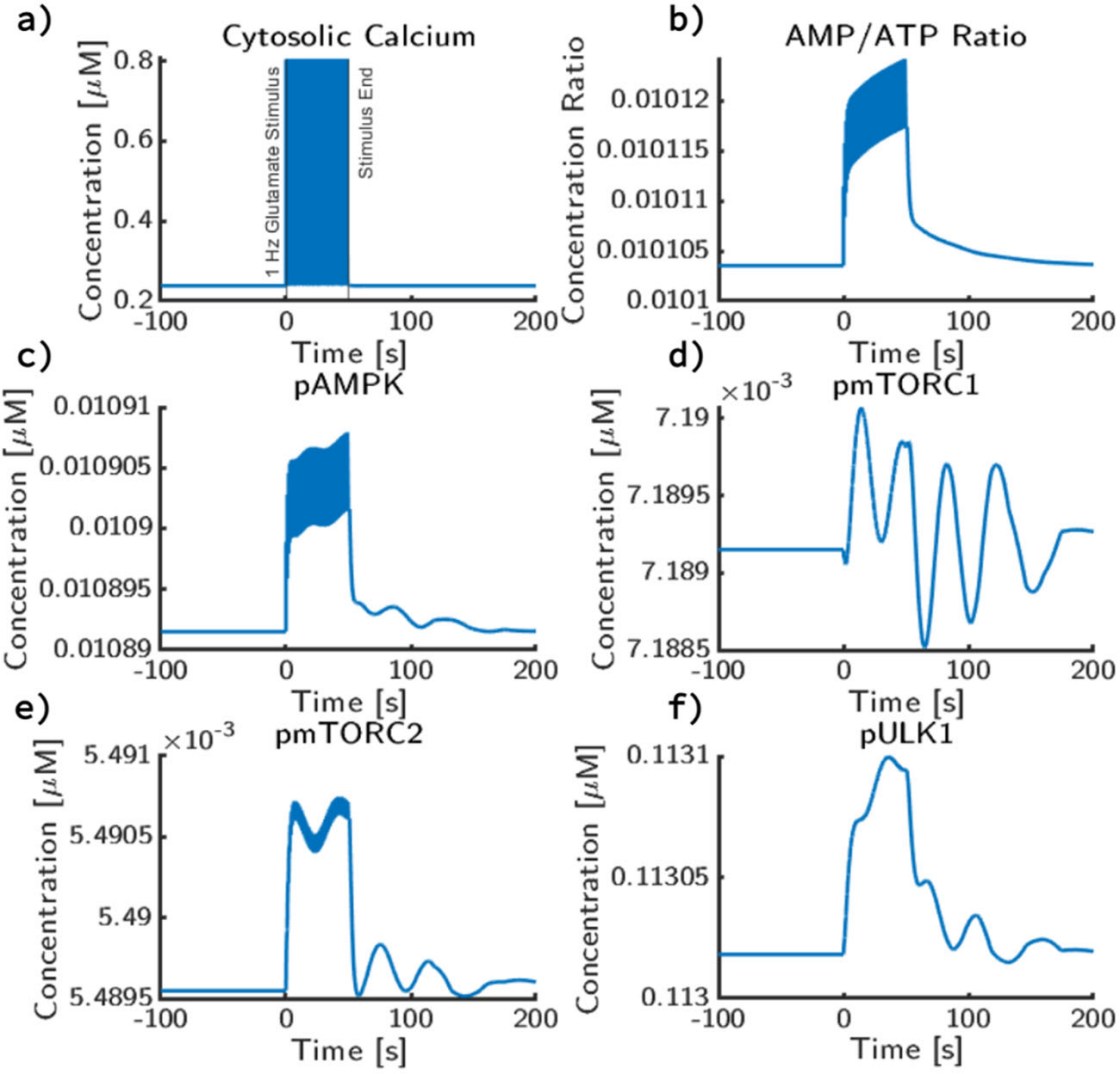
Effect of a simple 1 Hz glutamate on AMPK/mTORC dynamics. At *t* = 0s, a 1 Hz pulse train over 50 s is applied to the system. Before stimulus, the system was allowed to reach a steady state. During each pulse, 100 μM of glutamate is applied, which decays with a rate constant of 200 ms. After the pulse train, the system was allowed to return to an apparent steady state with no additional glutamate input. Concentration trajectories for **a** cytosolic calcium concentration, **b** cytosolic AMP/ATP ratio, **c** cytosolic phosphorylated AMPK concentration, **d** phosphorylated mTORC1 concentration, **e** phosphorylated mTORC2 concentration, **f** phosphorylated ULK1 concentration are shown.

After establishing the steady-state behavior in the absence of stimulus (Supplementary Figure 1), we perturb the system with a 1 Hz glutamate pulse for 50 seconds, which is representative of typical LTD induction protocol^48^. Glutamate stimulus induces a calcium influx (Fig. 3a) into the cytosol through the glutamatergic receptors, which consequently induces ATP consumption related to ion transport, increases the AMP/ATP ratio (Fig. 3b), and therefore AMPK activation. This AMPK activation (Fig. 3c) then leads to an initial increase of mTORC1 (Fig. 3d), mTORC2 (Fig. 3e), and ULK1 (Fig. 3f).

Cytosolic calcium attains a peak calcium concentration of 0.8 μM and decays rapidly to the baseline concentration of 0.2 μM (Fig. 3a). Due to the increased energy consumption associated with the calcium pumps, AMP/ATP ratio increases during synaptic signaling to a peak value of 0.01012 from its baseline value of 0.0101 (Fig. 3b). We note that both calcium and the AMP/ATP ratio (Fig. 3b) match the frequency of the input glutamate. pAMPK also shows a corresponding dynamic (Fig. 3c); phosphorylated AMPK increases by a marginal amount. However, instead of solely being influenced by glutamatergic signaling, AMPK also receives signals from the insulin system, thus has longer timescale oscillations. The magnitude of change for critical terms like mTORC1, mTORC2, and ULK1 are fairly small during 1 Hz stimulus of glutamate. In response to this AMPK activation, mTORC1 and mTORC2 both deviate a maximum of 0.5% but the trajectories observed are different; mTORC2 during stimulus reaches a higher peak value relative to its baseline oscillations while stimulus appears to lead mTORC1 to only slightly increase its amplitude (Fig. 3d, e). Changes in ULK1 follow similar trajectories to mTORC2 with a 1.2% change in activation decaying shortly after stimulus ends back to unstimulated values (Fig. 3f).

While the oscillation magnitudes are quite low in this case, it must be noted that the stimulus profile is most associated with LTD and therefore the energy demand and subsequent activation of downstream kinases of AMPK is expected to be low. However, this result is notable because it shows the crosstalk between two disparate signaling cascades through AMPK as a common vector. Through calcium and energy consumption, neuronal stimulus leads to direct changes in AMPK activation that influence the activity of mTORC1 and mTORC2. In the case of low-frequency stimulus and base parameter values, there is little influence, but these results suggest that signaling frequency may impact this crosstalk.

### Effect of glutamate stimulus frequency on AMPK/mTOR pathway

**Fig. 4:**
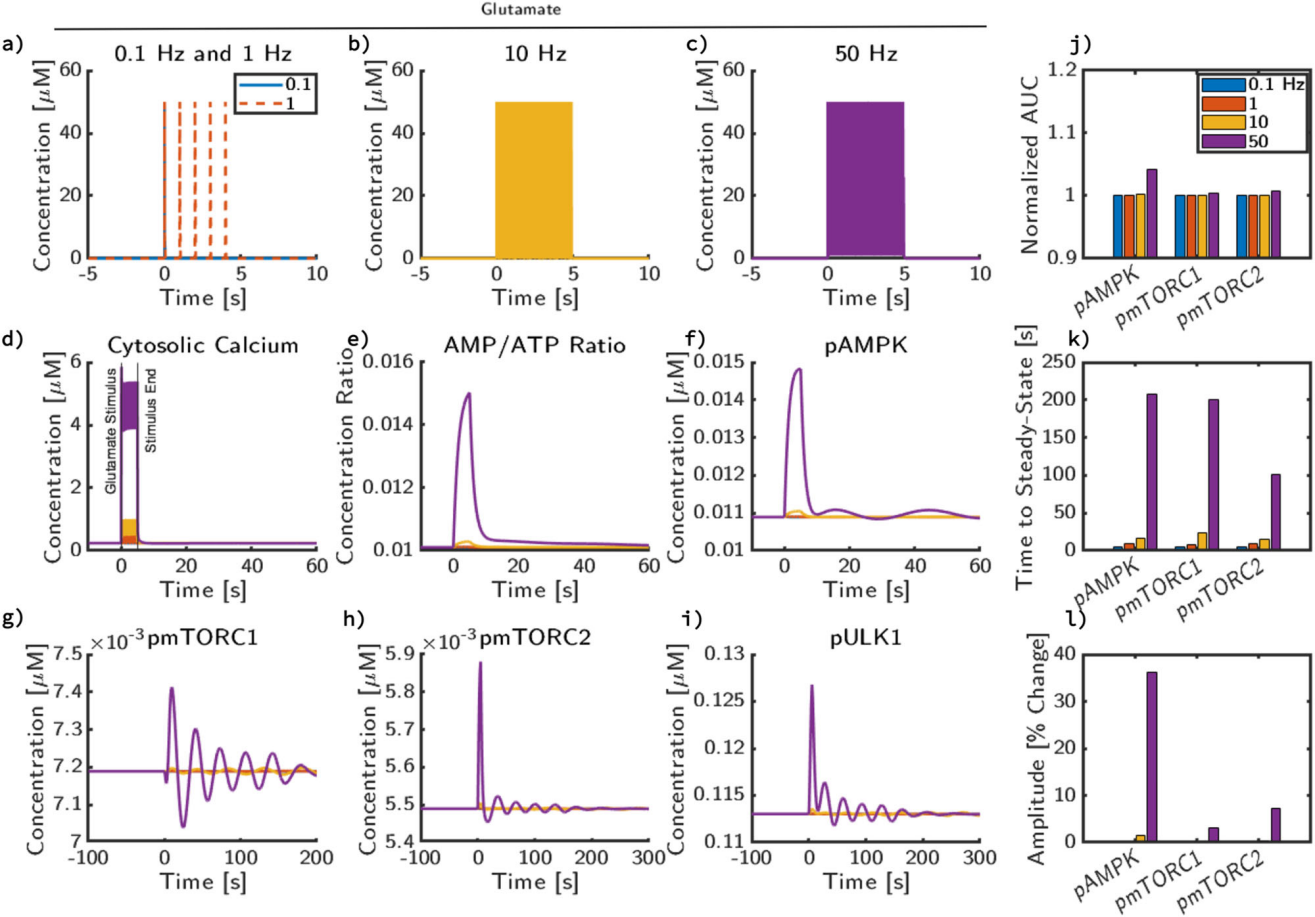
mTOR oscillations are stimulus frequency dependent. The system was stimulated with four different glutamate frequencies: **a** 0.1 Hz (blue), 1 Hz (orange), **b** 10 Hz (yellow), **c** 50 Hz (purple). After stimulus, simulations return to an apparent steady state with no additional glutamate input. Trajectories are shown for **d** cytosolic calcium concentration, **e** cytosolic AMP/ATP ratio, **f** phosphorylated AMPK concentration, **g** phosphorylated mTORC1 concentration, **h** phosphorylated mTORC2 concentration, **i** phosphorylated ULK1 concentration. Quantitative metrics for AMPK, mTORC1, and mTORC2 in response to pulse trains of glutamate stimulus are also shown: **j** Area under the curve (AUC) relative to the system without stimulus applied over the same integration window (100 s), **k** time to reach steady state, and **l** amplitude change quantified as percent change from steady-state value.

We next investigated the effect of glutamate stimulus frequency on the activation profiles of key model outputs. The glutamate input stimuli, shown in Fig. 4a–c, was varied from 0.1 Hz to 50 Hz, reflective of the range of potential stimulus frequencies in a signaling neuron^47^. While in Fig. 3, we show only minor changes in state and overall signaling effect, as stimulus frequency increases, cytosolic calcium concentration, and ATP consumption also increase (Fig. 4d, e). The change in calcium concentration is dependent on the frequency of glutamate stimulus (Fig. 4d); as stimulus frequency increases, calcium amplitude increases and the higher frequencies are filtered out (also see refs. ^2,49^). There is a corresponding ATP consumption that leads to an increase in the AMP/ATP ratio (Fig. 4e), which leads to AMPK activation (Fig. 4f). In low levels of stimulus frequency, mTORC1 (Fig. 4g) and mTORC2 (Fig. 4h) oscillate around the baseline value. When AMPK is activated in higher magnitudes, we observe a large initial perturbation from the baseline oscillatory pattern that results in damped oscillations that eventually return to steady-state concentrations. The maximum amplitude of the deviations from steady state of mTORC1, mTORC2, and ULK1 (Fig. 4g–i) are dependent on the amplitude of AMPK activation, and therefore glutamate stimulus frequency. Increasing stimulus frequency does not appear to significantly lead to a phase shift in frequency for any species, but most notably increases the initial magnitude of oscillations.

The AUC over 100 s (Fig. 4j) represents the total amount of a species within the system during short-term response to signaling pulse trains. Since the concentrations of each species can be orders of magnitude apart, we normalized each AUC to the baseline activity profiles to obtain a relative AUC value. In Fig. 4j, we observed a frequency-dependent increase in the AUC of AMPK. However, for mTORC1 and mTORC2, only very minor increases in AUC were observed, 1% and 2%, respectively. At low-frequency conditions (0.1 and 1 Hz), the AUC of all species does not change significantly. As frequency increases, AMPK activation increases proportionally to the highest stimulus frequency, where AMPK’s AUC increases by 5% after exposure to 50 Hz stimulus for 5 s. While the overall magnitude of change for mTORC1’s AUC is lower in comparison to pAMPK and mTORC2 for all frequencies, there is a slight increase as frequency increases.

Next, in Fig. 4k and Fig. 4l, we characterized the damped oscillatory behavior of the trajectories through its time to steady state and amplitude. All three species have a similar trend in which higher frequencies have longer times to steady state (TTS) and higher amplitudes. Another point of note is that the increase in TTS with respect to frequency is consistent up to 10 Hz, but 50 Hz shows a significant disparity between AMPK, mTORC1, and mTORC2. Both AMPK and mTORC1 have TTS of around 200 seconds, while mTORC2 returns to steady state in 100 s. As for amplitudes (Fig. 4l), AMPK maximum amplitude is significantly higher due to direct activation from AMP/ATP ratio, a 36% increase in amplitude during 50 Hz stimulus compared to 1 Hz stimulus. mTORC1 has the lowest increase in amplitude due to high-frequency stimulus, ~3% increase compared to steady state. The maximum amplitude for mTORC2 is higher than mTORC1, but even at 50 Hz, only 7.5% increase over steady state. Overall, there is a separation between high-frequency and low-frequency stimulus in the observed trajectories and metrics. While the predicted AUC, TTS, and amplitude are similar between 0.1 and 1 Hz stimulus, the model predicts up to a 5% increase in AUC for high-frequency stimulus and a significant increase in observed oscillation duration.

### Increasing basal energy consumption increases AMPK activation

While calcium and other ion transport are known to be primary drivers of energy consumption in neurons, there are additional energy-consuming processes that require ATP in the dendritic spine. For example, it is thought that actin, which is abundant in dendritic spines, is one of the main non-signaling ATP sinks through actin polymerization and remodeling^50^. In addition, protein and lipid turnover in neurons can consume up to 25% of the total ATP consumed in brain tissue^14,51^.

To explore the interaction between the basal energy consumption rate and the glutamate stimulus on AMPK signaling, we designed a series of simulations using our model. In our model, energy consumption is simplified as a lumped parameter representing ATP hydrolysis in the cytosol, *k*_*hyd*_. We varied this parameter to capture the effect of different energy consumption rates. We held our glutamate stimulus to 10 Hz because this stimulus approximates the threshold of frequency necessary to trigger change in mTOR (Fig. 4). In this set of simulations, all variations had the same set of initial conditions, but at *t* = 0, *k*_*hyd*_ was changed in addition to glutamate pulse trains of 10 Hz.

**Fig. 5:**
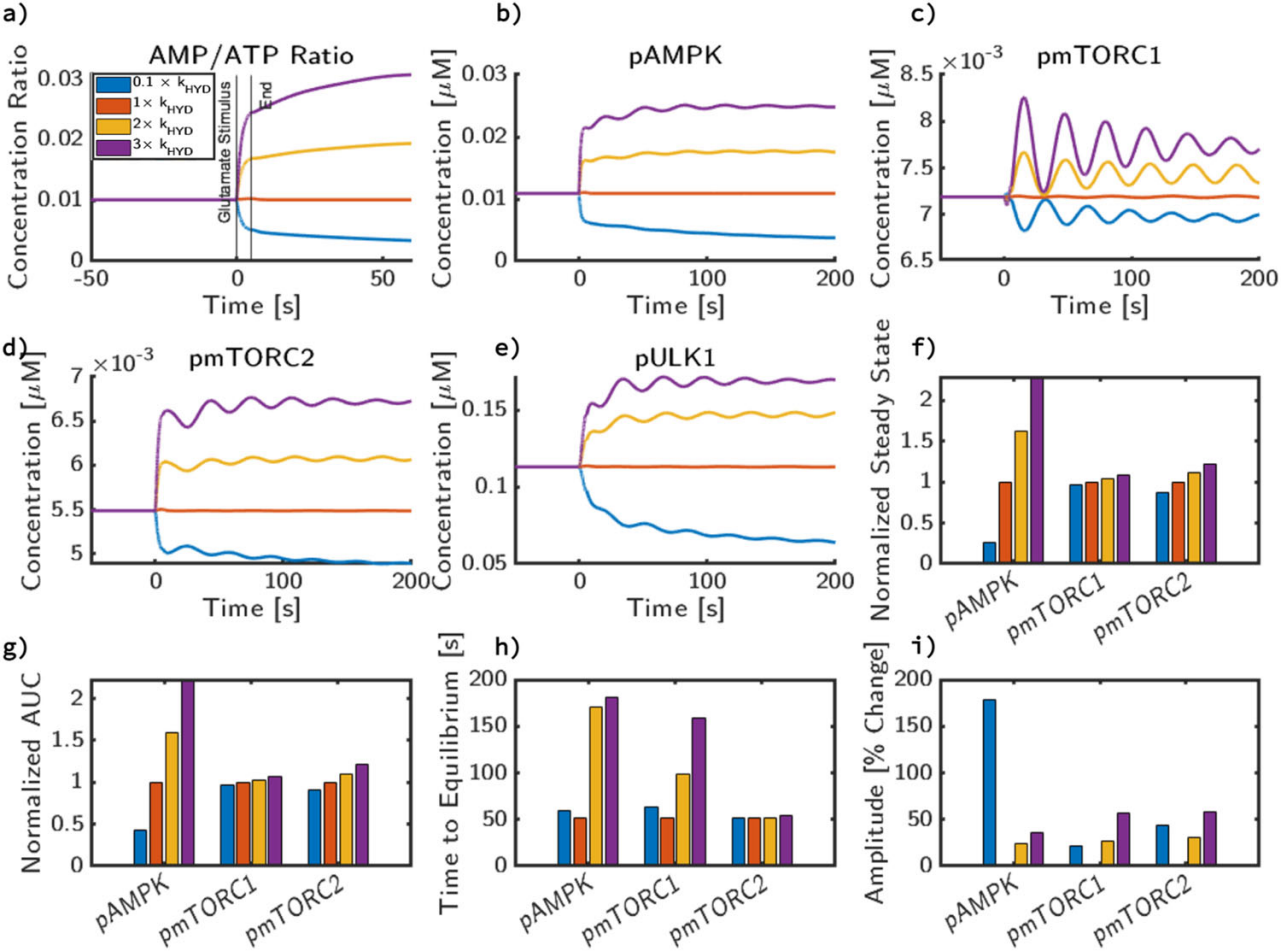
Cellular metabolic rate influences steady-state behavior of AMPK and mTOR independent of calcium. We apply a 10 Hz glutamate stimulus for 5 s, however, at *t* = 0 s, we also change the value of baseline energy consumption throughout the cell and plot concentration trajectories for **a** AMP/ATP, **b** active, phosphorylated AMPK, **c** active, phosphorylated mTORC1, **d** active, phosphorylated mTORC2, **e** active, phosphorylated pULK1. Additionally, in (**f**), we compare the changes in steady state for pAMPK, mTORC1, and mTORC2 with respect to changes in hydrolysis rate. In (**g**), we then compare how this change impacts AUC, normalized to the AUC of simulation with the base value of the parameter. In (**h**), we plot the time to reach steady state and in (**i**), the relative magnitude of the first peak of AMPK, mTORC1, and mTORC2 to its new steady-state value as a result of energy consumption from glutamatergic stimulus.

We found that varying *k*_*hyd*_ had led to direct changes in the peak and steady-state behavior of AMP/ATP ratio (Fig. 5a). There was no change in calcium dynamics since the calcium influx depends on the NMDAR fluxes, which are not dependent on hydrolysis rates. Compared to the baseline value, a 10-fold decrease in *k*_*hyd*_ decreases the AMP/ATP ratio by a small amount but a 3-fold increase in *k*_*hyd*_ increases AMP/ATP ratio dramatically. This behavior is reflected in pAMPK (Fig. 5b), where a nonlinear increase in pAMPK levels is seen for a 3-fold increase in the energy consumption rate. Interestingly, changing *k*_*hyd*_ actually changes the oscillation pattern for pmTORC1 (Fig. 5c). Increase in the energy consumption rate translates the damped oscillations observed in pmTORC1 at lower energy consumption rates into a stable steady state, without any oscillations, at high energy consumption rates. This effect is also seen in the dynamics of pmTORC2 (Fig. 5d) and pULK1 (Fig. 5e) except that increasing basal energy consumption increases the level of pmTORC2 and pULK1. Further analysis of these variations reveals that the steady state depends on the basal energy consumption (Fig. 5f).

Next, we characterize the effect of hydrolysis rates on the dynamic features of AMPK, mTORC1, and mTORC2 signaling by plotting the normalized AUC, TTS, and percent amplitude (Fig. 5g–i). For AMPK phosphorylation, there is an increase in AUC with increasing *k*_*hyd*_. From the baseline value of *k*_*hyd*_, reducing *k*_*hyd*_ to a tenth of its original value roughly produces an AUC of half. However, the increase of *k*_*hyd*_ to 2 × *k*_*hyd*_ and 3 × *k*_*hyd*_ increases the relative AUC by 50% and 120%, respectively. For mTORC1, while the increase in AUC with increasing *k*_*hyd*_ is apparent, the magnitude of increase is not as high when compared to AMPK and is within a few percent of the steady-state value. This trend can qualitatively be observed in Fig. 5c, as the variations of *k*_*hyd*_ from 0.1 to 2 × *k*_*hyd*_ have roughly the same trajectories, but the variation to 3 × *k*_*hyd*_ increases the steady state substantially. For mTORC2, the AUC increase from 0.1 to 1 × *k*_*hyd*_ is minor relative to the change in *k*_*hyd*_, however 3 × *k*_*hyd*_ produces a 20% jump in AUC. 2 × *k*_*hyd*_ and 3 × *k*_*hyd*_ both have proportional responses relative to the change in *k*_*hyd*_.

Next, the time to steady-state of AMPK (Fig. 5h) increases slightly with both increasing and decreasing *k*_*hyd*_ from baseline value. This is surprising, because for most metrics there appears to be a consistent trend following hydrolysis rate. The mTORC1 and mTORC2 TTS both follow similar trends of monotonic increase relative to *k*_*hyd*_. This could be caused by the 0.1 × *k*_*hyd*_ steady state (blue curve) being further away from the initial state than the 1 × *k*_*hyd*_ case (represented by the orange curve).

Finally, the amplitude change (Fig. 5i) depends on the magnitude of *k*_*hyd*_ since, as shown in Fig. 5f, the steady state is dependent on *k*_*hyd*_. Simulations with high changes in *k*_*hyd*_ had the highest percent change in amplitude due to the initial equilibration. In general, the 0.1 × *k*_*hyd*_ and 3 × *k*_*hyd*_ simulations produced the highest percent change relative to their individual steady state. Thus, we found that the stability features of the AMPK/mTOR pathway depend on the basal rate of ATP hydrolysis—increasing ATP consumption allows the system to transition from an oscillatory state to a stable steady state.

### Insulin signaling and metabolic signaling interact to govern stability features

Thus far, we have explored the impact of glutamate stimulus and energy consumption on AMPK and mTOR activation. We next investigated the role of the insulin response pathway and its crosstalk with the glutamate pathway. Insulin receptor signaling is a primary input in metabolism and influences the phosphorylation state of AMPK, mTORC1, and mTORC2. In the model originally developed by Sadria^29^, the insulin receptor substrate (IRS) directly interacts with AMPK and mTORC2, resulting in regimes of oscillations dependent on the rate of IRS phosphorylation. Since we included this pathway in our model, we now explore the feedback between the IRS pathway and AMPK pathway through the energy consumption rate. In our model, the parameters controlling the IRS phosphorylation rate and energy consumption rate are, V_*IR*_ and *k*_*hyd*_, respectively. In Fig. 2c–e, parameters corresponding to V_*IR*_ and *k*_*hyd*_ had significant control over the steady-state concentrations of AMPK, mTORC1, and mTORC2 under no glutamate stimulus. Here, we investigated how V_*IR*_, *k*_*hyd*_, in conjunction with glutamate stimulus impact the AMPK-mTOR pathway.

**Fig. 6:**
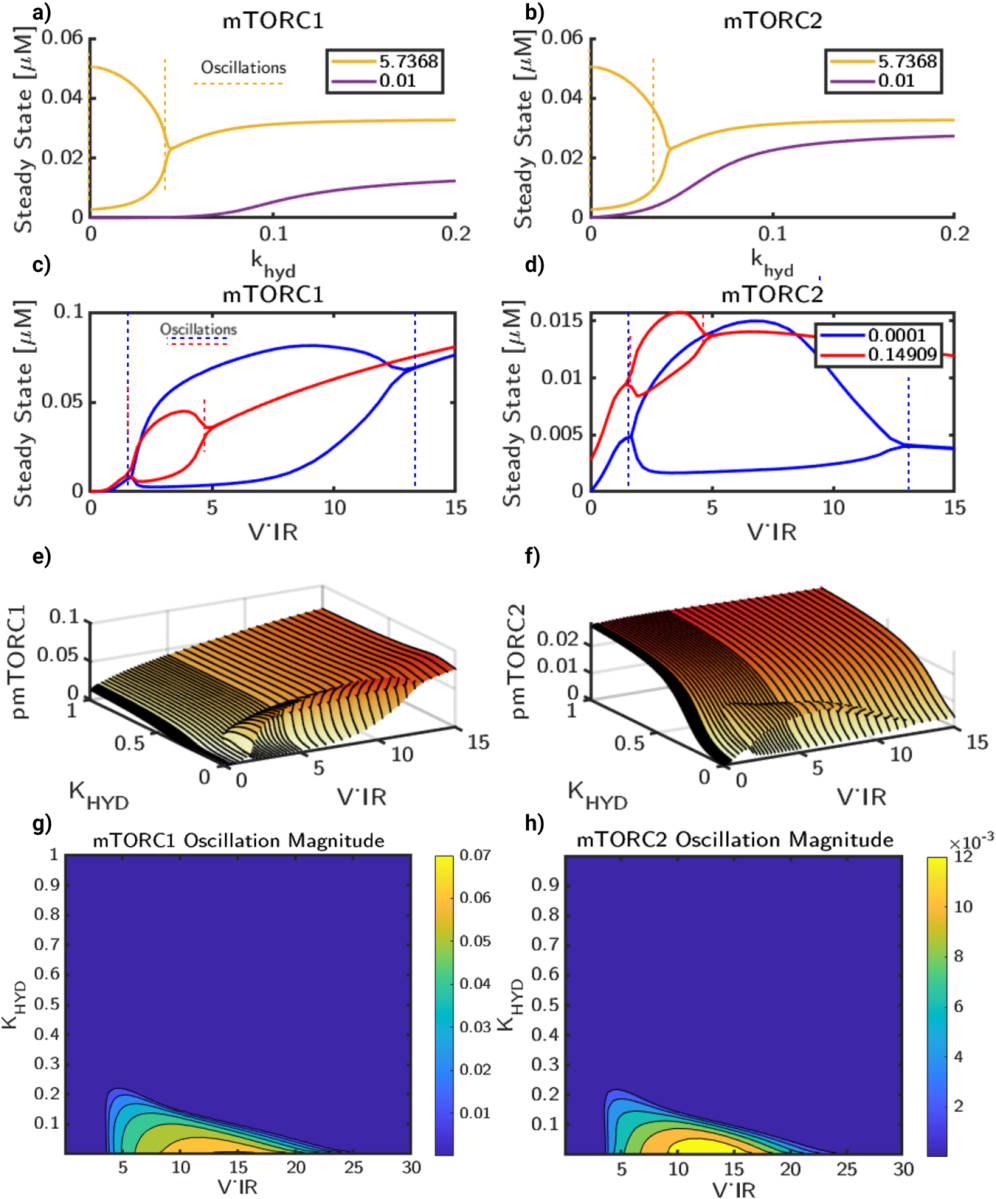
Oscillatory regimes for mTOR activation are dependent on both metabolic activity and IRS. The system displays oscillatory behavior dependent on both *k*_*hyd*_ and V_*IR*_ rates. We show the dependence of **a** mTORC1 and **b** mTORC2 stability on *k*_*hyd*_ by selecting two values of V_*IR*_, 5.7368 [mM/s] (yellow) and 0.01 [mM/s] (purple). For each case, we simulate until an apparent steady state for a range of *k*_*hyd*_ and plot the curves of minima and maxima at steady state. In the oscillatory region, the minima and maxima diverge and show an oscillatory regime, denoted by yellow dashed line. Next, we hold *k*_*hyd*_ constant and vary V_*IR*_ to obtain the stability profiles for **c** mTORC1 and **d** mTORC2 dependent on V_*IR*_ For two values of *k*_*hyd*_, 1 × 10^−4^ [mM/s] and 0.149 [mM/s] we simulate until an apparent steady state for a range of V_*IR*_, then plot the curves of local minima and maxima at steady state for mTORC1 and mTORC2. For regions of monostability, outside of dashed lines, the curves of minima and maxima converge to the steady state. In oscillatory regimes, within the dashed lines, the local minima and maxima due to oscillations form an envelope. The size and shape of the envelope is dependent on both *k*_*hyd*_ and V_*IR*_. Then, to characterize this relation in a 2D parameter space, we plot corresponding 3D surface plots for **e** mTORC1 and **f** mTORC2. We also plot the oscillation magnitudes for **g** mTORC1 and **h** mTORC2, showing the parameter space that results in sustained oscillations.

In Fig. 6, we show how the stability of the model is dependent on the magnitude of *k*_*hyd*_ and V_*IR*_. First, for hydrolysis (Fig. 6a, b), we select two values of V_*IR*_ to hold constant (5.7368 mM/s yellow and 0.01 mM/s in purple) and vary the rates of *k*_*hyd*_ (1000 values between 1 × 10^−4^ to 1 mM/s). We simulate the system in the absence stimulus until steady state for each parameter combination and compute the minima and maxima as a function of V_*IR*_. We observed that for the lower value of V_*IR*_, increasing *k*_*hyd*_ gives a single unique steady state for both mTORC1 and mTORC2 (Fig. 6a, b). For higher values of V_*IR*_, we found that the stability behavior has an oscillatory regime for low values of *k*_*hyd*_ and a single steady state for high values of *k*_*hyd*_. Thus, the hydrolysis rate alone can alter the stability features of the coupled system. Next, to obtain the relation between stability and V_*IR*_ values, we select two values of *k*_*hyd*_ and vary with 60 values of V_*IR*_ (from 0.1 to 20 mM/s), shown in Fig. 6c, d. In Fig. 6c, we compute the minima and maxima as a function of V_*IR*_ to show the stability features for two values of *k*_*hyd*_, 0.0001 (blue) and 0.149 (red) [mM/s]. For low and high values of V_*IR*_, minima and maxima curves converge to a singular value. However, there is a range of V_*IR*_ values in which the curves diverge. In this range, we see oscillations similar to those seen in the steady state of Fig. 3c–f. Values of V_*IR*_ that result in oscillatory behavior are correlated with hydrolysis; as *k*_*hyd*_ increases, a smaller range of V_*IR*_ produces oscillations. Compared to a low value of *k*_*hyd*_ (blue, 0.0001 [mM/s]), a high value of *k*_*hyd*_ (red, 0.149 [mM/s]) shows a significantly smaller range of values, as well as a much smaller range of oscillations. This same approach is taken with Fig. 6d for mTORC2.

Finally, we varied both *k*_*hyd*_ and V_*IR*_ and plot the same curves as surface plots in 3D. For mTORC1 (Fig. 6e), rising V_*IR*_ leads to an increase in the steady state. Furthermore, increasing *k*_*hyd*_ reduces the range of values that produce oscillatory behavior. Outside the oscillatory regime, mTORC1 has a monotonic relationship with V_*IR*_. Within the parameter range studied, the steady-state behavior of mTORC1 is strongly related to the magnitude of V_*IR*_, while *k*_*hyd*_ has diminishing impact past the oscillatory envelope. For mTORC2 (Fig. 6f), a similar regime of oscillations was found. However, for mTORC2, V_*IR*_ does not strongly affect the magnitude of mTORC2 at steady state. In contrast to mTORC1, in which V_*IR*_ was the primary controller of mTORC1 steady state, *k*_*hyd*_ results in a much stronger increase mTORC2 steady state for all values of V_*IR*_. Corresponding contour plots highlight the oscillatory regimes for mTORC1 (Fig. 6g) and mTORC2 (Fig. 6h). In summary, the oscillations of mTORC1 and mTORC2 that are observed in the model are dependent on the values of *k*_*hyd*_ and V_*IR*_ and we predict that high energy consumption states (high values of *k*_*hyd*_) result in monostable steady states, while low values of *k*_*hyd*_ and enable steady-state oscillations within a range of V_*IR*_ values.

#### Signaling frequency, ATP consumption, and insulin signaling modulate system response to energy stress

**Fig. 7:**
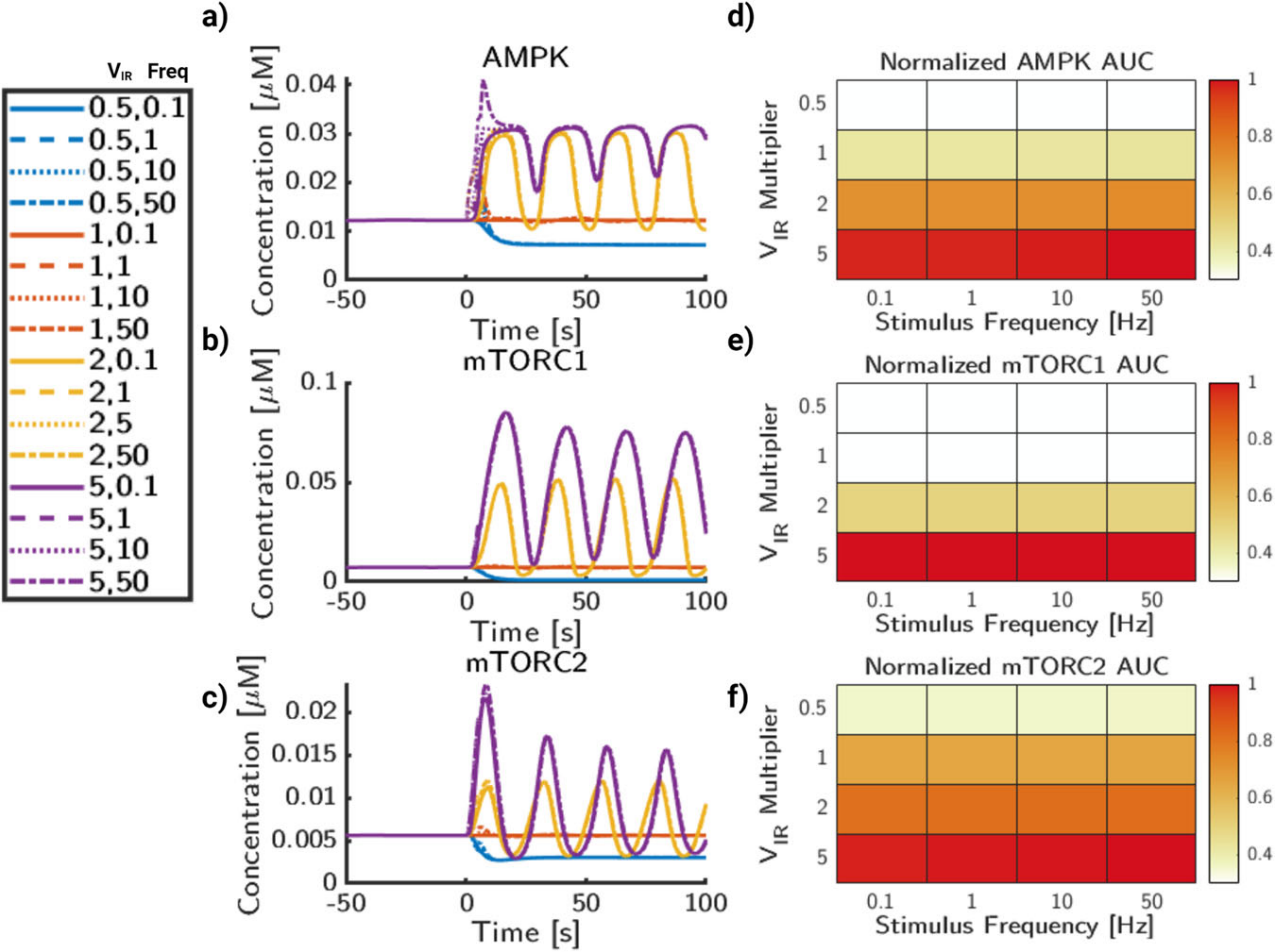
Insulin and cellular metabolism govern phosphorylation rates of AMPK, mTORC1, and mTORC2. **a** AMPK phosphorylation concentration trajectories for a range of values for insulin receptor activity, V_*IR*_, and ATP consumption rates, *k*_*hyd*_. **b** mTORC1 phosphorylation concentration trajectories for a range of values for insulin receptor activity, V_*IR*_, and ATP consumption rates, *k*_*hyd*_. **c** mTORC2 phosphorylation concentration trajectories for a range of values for insulin receptor activity, V_*IR*_, and ATP consumption rates, *k*_*hyd*_. **d** AMPK AUC normalized to the maximum AUC value of AMPK. **e** mTORC1 AUC normalized to the maximum AUC value of mTORC1. **f** mTORC2 AUC normalized to the maximum AUC value of mTORC1. Color for heatmaps are AUC relative to maximal AUC per graph.

Thus far, we have investigated the impact of glutamate frequency alone (Fig. 4) and the bifurcation behavior of the metabolic pathways in Fig. 6. Next, we show glutamate stimulus frequency, IRS, and hydrolysis rate combine to influence the energy state of the system. In Fig. 7, we observe the trends associated with changes in V_*IR*_ and *k*_*hyd*_ under a 10 Hz stimulus pulse for 5 s. Trajectories for pAMPK, mTORC1, and mTORC2 are shown in Fig. 7a–c. In these trajectories, the line color refers to the magnitude of V_*IR*_ while the line markers represent the *k*_*hyd*_ magnitude. Qualitatively, increases in *k*_*hyd*_ correspond to small concentration shifts of the trajectories predicted by V_*IR*_. For clarity, we will refer to the parameter values relative to the base model values (V_*IR*,*b*_ and *k*_*hyd*,*b*_). Concentration trajectories for AMPK are shown in Fig. 7a. We observed that for most trajectories, the peak activity corresponds with the time of the stimulus. In addition, for the case of V_*IR*_ = 10 × V_*IR*,*b*_ (purple lines) for AMPK, the rate of *k*_*hyd*_ appears to affect the phase of the oscillations as well. Interestingly, this does not appear to be reflected in any other value of V_*IR*_ as the only oscillations of large magnitude at this frequency of stimulus appear to be due to V_*IR*_.

For mTORC1 (Fig. 7b), we see a similar trend in the curves generated by increasing V_*IR*_ values to 10 × V_*IR*,*b*_. While many of the curves have a relatively low steady state and transient concentration as a result of the 10 Hz stimulus, The phase shift observed with AMPK in Fig. 7a also leads to a shift in phase for mTORC1, however, the steady state of these curves and direction of the oscillations shifts. This is exemplified most by 1 × *k*_*hyd*,*b*_ and 2 × *k*_*hyd*,*b*_, in which the relative size magnitudes of oscillations are similar in magnitude, however the initial steady state is different. Furthermore, oscillations are abrogated by higher values of *k*_*hyd*_, which is consistent with the higher non-oscillatory regime of mTORC1 found in Fig. 6e. For mTORC2 (Fig. 7c), there are a spectrum of states available to mTORC2 phosphorylation dependent on *k*_*hyd*_, but more strongly on V_*IR*_ value. Similar to mTORC1, mTORC2 has a phase shift in the oscillations, but not necessarily the direction of the oscillations as the steady states are more similar between 0.1, 1, and 2 × *k*_*hyd*,*b*_.

The relative AUC of the trajectories in Fig. 7a–c are then quantified and summarized in Fig. 7d–f. Since the curves can be vastly different in magnitude, for ease of comparability all AUC’s are ratios of the AUC of an unstimulated system under the same integration duration and then normalized to the maximum AUC for the selected trajectories. For AMPK (Fig. 7d), increasing V_*IR*_ and *k*_*hyd*_ both lead to increases in AUC, with larger increases correlated to higher magnitudes of *k*_*hyd*_. For mTORC1 (Fig. 7e), increasing V_*IR*_ increases mTORC1 AUC, but increasing *k*_*hyd*_ leads to slight increase in mTORC1 AUC. Finally, for mTORC2 (Fig. 7f), the AUC for most of the trajectories are similar to AMPK. However, the trend follows that increasing both V_*IR*_ and *k*_*hyd*_ also increases the mTORC2 AUC, and perhaps mTORC2 has the highest sensitivity to *k*_*hyd*_. The largest magnitude increase of mTORC2 activation comes from when both *k*_*hyd*_ and V_*IR*_ are highest.

**Fig. 8:**
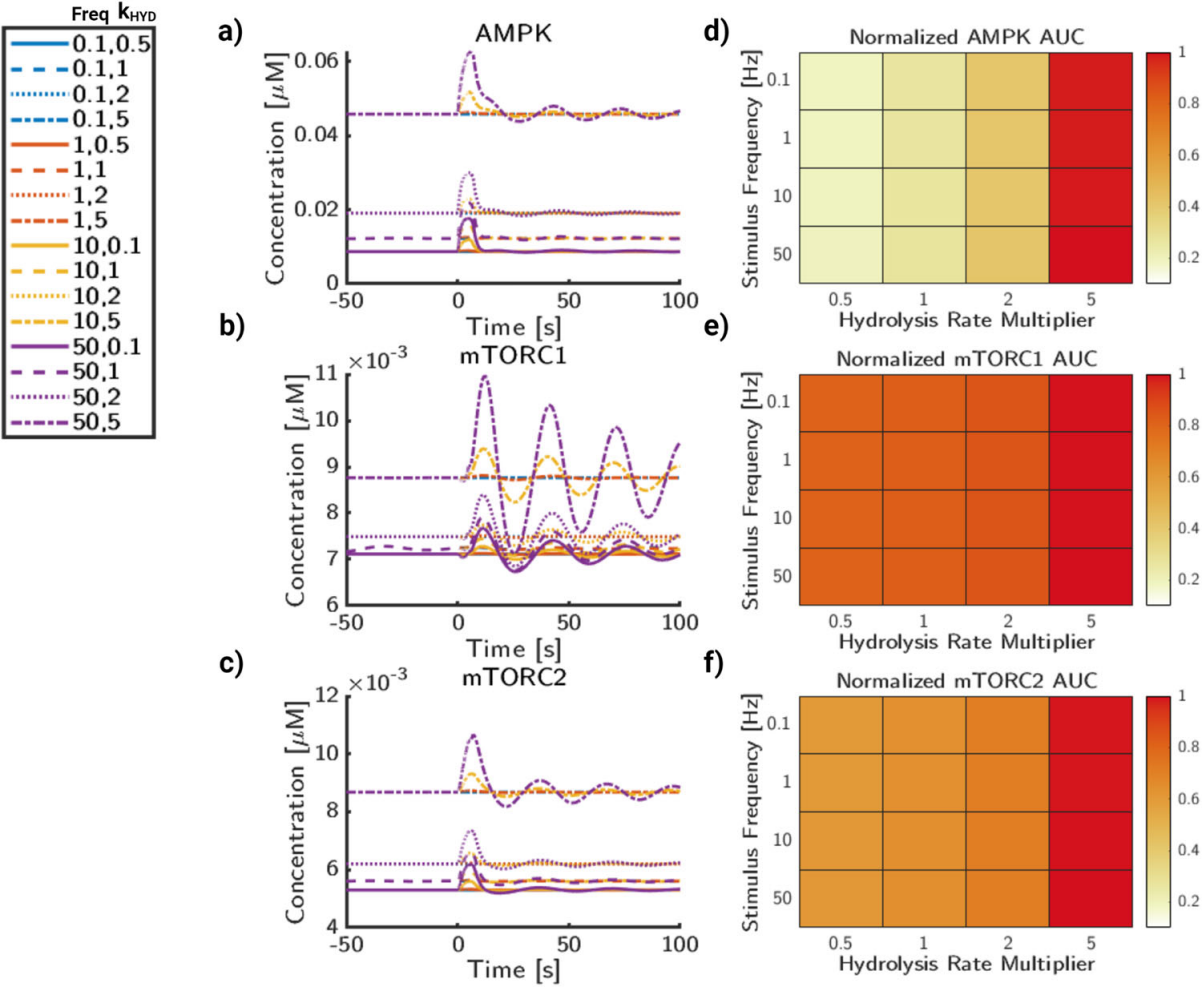
Insulin signaling and glutamate frequency influence dynamic behavior of AMPK and mTOR phosphorylation. **a** AMPK phosphorylation concentration trajectories for a range of values for insulin receptor activity and glutamate stimulus frequency. **b** mTORC1 phosphorylation concentration trajectories for a range of values for insulin receptor activity and glutamate stimulus frequency. **c** mTORC2 phosphorylation concentration trajectories for a range of values for insulin receptor activity and glutamate stimulus frequency. **d** AUC for AMPK normalized to the maximum AUC value of AMPK. **e** AUC for mTORC2 normalized to the maximum AUC value of mTORC1. **f** AUC for mTORC2 normalized to the maximum AUC value of mTORC2. Color for heatmaps are AUC relative to maximal AUC per graph.

We next analyzed the interaction of the insulin substrate strength, V_*IR*_, with the glutamate stimulus frequency (Fig. 8). V_*IR*_ influences the steady state and oscillatory values of AMPK activity (Fig. 8a), which does not appear to be as strongly impacted by glutamate frequency on this concentration scale. But, as shown previously in Fig. 4, higher frequencies above 10 Hz induce large transient pulses of AMPK activity. The primary influence of frequency appears to be increasing the activity of AMPK during stimulus. mTORC1 (Fig. 8b) is significantly more sensitive to the magnitude of V_*IR*_ than to the input frequency. This is the case for the high-frequency cases of both 0.1 and 10 × V_*IR*_, in which no dampened oscillations are shown after approximately 50 seconds. A similar case is observed with mTORC2 (Fig. 8c), in which both V_*IR*_ and stimulus frequency impact the initial peak height. However, particularly at cases of high frequency and also high V_*IR*_ magnitude, there are high amplitude oscillations and a delayed return to steady state. Oscillations are still observed in 1 and 2 × V_*IR*_ cases, but are less pronounced due to the wider range of magnitudes from higher frequencies.

**Fig. 9:**
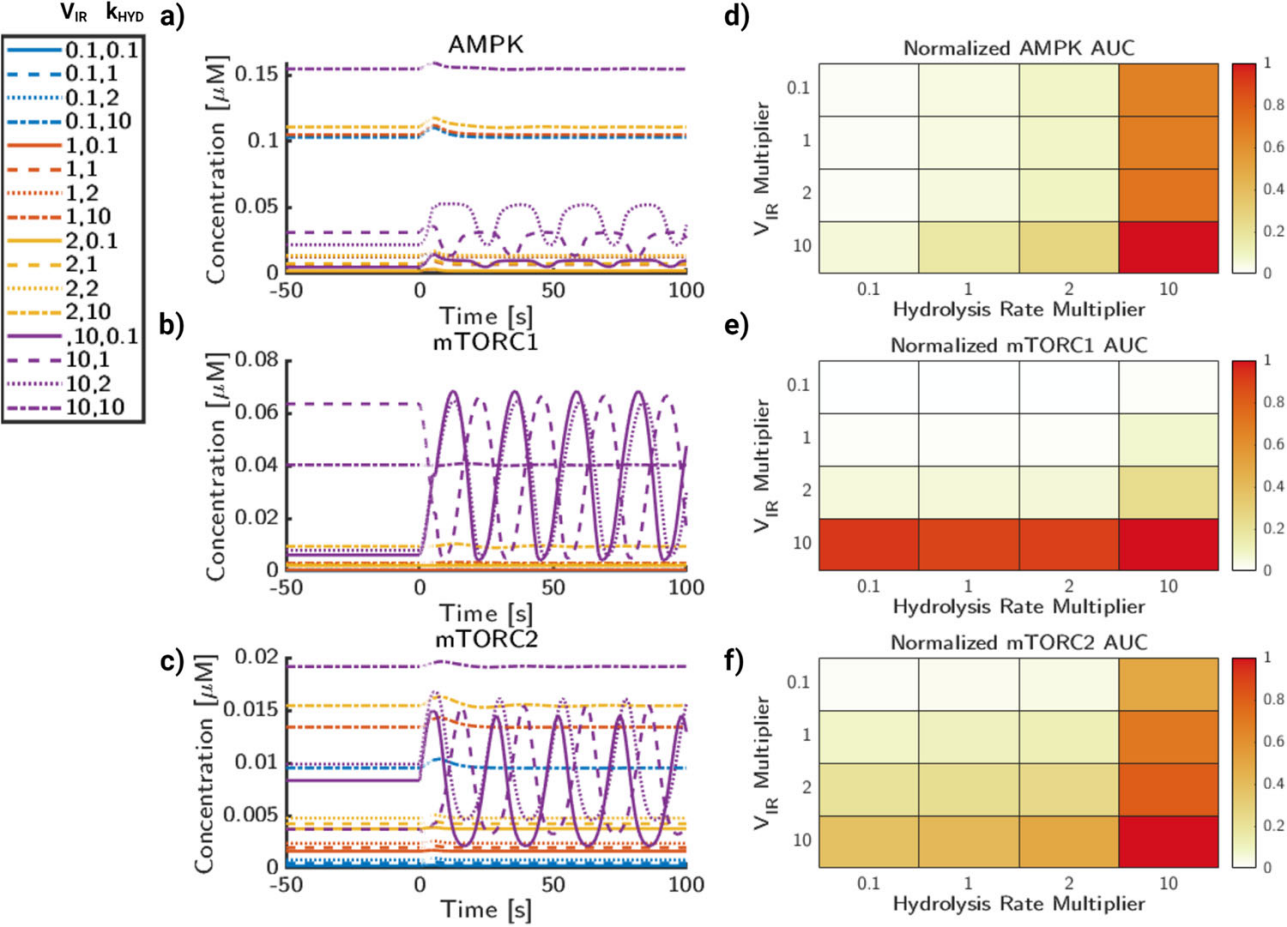
Glutamate frequency and metabolic stress lead to increases in AMPK and mTOR deviations. **a** AMPK phosphorylation trajectories for a range of values for glutamate frequency and ATP consumption rates, *k*_*hyd*_. **b** mTORC1 phosphorylation trajectories for a range of values for glutamate stimulus frequency and ATP consumption rates, *k*_*hyd*_. **c** mTORC2 phosphorylation trajectories for a range of values for glutamate stimulus frequency and ATP consumption rates, *k*_*hyd*_. **d** AUC for AMPK normalized to maximal AUC value. **e** AUC for mTORC1 normalized to maximal AUC value. **f** AUC for mTORC2 normalized to maximal AUC value. Color for heatmaps are AUC relative to maximal AUC per graph.

The relative AUC (Fig. 8d–f) is quantified in the same process as Fig. 7. For AMPK (Fig. 8d), the relative AUC increases more with VIR than stimulus frequency. The highest magnitude of AUC for AMPK comes from both high stimulus frequency as well as high V_*IR*_ magnitude. For mTORC1 (Fig. 8e), while there is a clear increase in AUC while V_*IR*_ magnitude increases, any differences due to frequency are minimal. This implies that the stimulus frequency does not impact the overall signaling capacity of mTORC1, but may impact the dynamics of signaling on short timescales. Likewise, mTORC2 (Fig. 8f) similarly shows a dependence on V_*IR*_ magnitude for overall mTORC2 AUC. However, as frequency increases, the AUC increases, but at a lower rate than changing the insulin receptor signaling strength. Finally, we analyze interactions between signaling frequency and ATP consumption rate (Fig. 9). As before, stimulus frequency appears to be the largest driver of transient stimulus for AMPK (Fig. 9a). As frequency increases, the time AMPK returns back to steady state is delayed, especially for higher magnitudes of *k*_*hyd*_. The rate of *k*_*hyd*_ can also significantly raise the baseline values of AMPK activation, which is consistent with its model function and AMPK’s activation conditions as the AMP/ATP ratio increases with higher *k*_*hyd*_. For mTORC1 (Fig. 9b), *k*_*hyd*_ does not have a strong impact to the activation rates of mTORC1, except at very high rates of *k*_*hyd*_. The stimulus frequency, however, has a strong correlation with increased amplitude of mTOR1. For mTORC2 (Fig. 9c), frequency again shows an increased transient amplitude increase, but not a significant change to the overall trajectories. Likewise, *k*_*hyd*_ only appears to significantly change the state of mTORC2 when it is very high, 10 × *k*_*hyd*,*b*_.

The relative AUC (Fig. 9d–f) is quantified in the same process as Fig. 7. For AMPK (Fig. 9d), the relative AMPK AUC shows a strong dependence on *k*_*hyd*_, but also a minor increase due to frequency. For mTORC1 (Fig. 9e), frequency changes appear to affect the dynamics of the system, but not necessarily the AUC. While the initial peak heights increase, this does not appear to impact the overall signaling capacity of the system. However, this may lead to differences in short timescale activation profiles of mTOR and its downstream targets. For mTORC2 (Fig. 9f), the AUC shows an overall increase with hydrolysis rate. It also displays a minor increase due to signaling frequency, particularly at higher hydrolysis rates. In summary, we have shown that crosstalk between glutamate frequency and metabolic signaling plays an important role in protein kinase activity, which has implications on cell fate and neuronal function. Glutamate signaling frequency appears to control the dynamic behavior, including the amplitude change and observed oscillations, however, cellular energy consumption controls the steady state, which has a much stronger influence over AUC magnitude.

## Discussion

In this study, we have investigated the crosstalk between calcium influx in dendritic spines and mTOR and AMPK activation bridging multiple timescales of signaling in the context of dendritic spines using computational modeling. The pathways explored here have significant implications for synaptic plasticity as the downstream pathways of mTOR have been shown to be necessary for LTP and LTD^34^. Our main predictions focus on the possible divergent stability regimes in the system as a function of crosstalk between signaling and metabolic pathways. We discovered that the model displays a parameter-driven oscillatory pattern This allows neurons to adjust their cellular response to stimuli based on energy usage, external factors such as insulin signaling, and the frequency of the signals received.

It is believed that synaptic plasticity is triggered mainly through high-frequency signaling^46^. While the exact mechanisms contributing to synaptic plasticity are unknown, various works have shown that it is dependent on subcellular structure, calcium signaling, metabolics, and many other intermediary proteins including mTOR^4,47,52,53^. In addition, the mechanisms contributing to synaptic pruning and loss of dendritic spine density are unknown. In such complex processes, mathematical modeling may be able to contribute strongly to our understanding of crosstalk between signaling, metabolism, and synaptic plasticity.

**Fig. 10:**
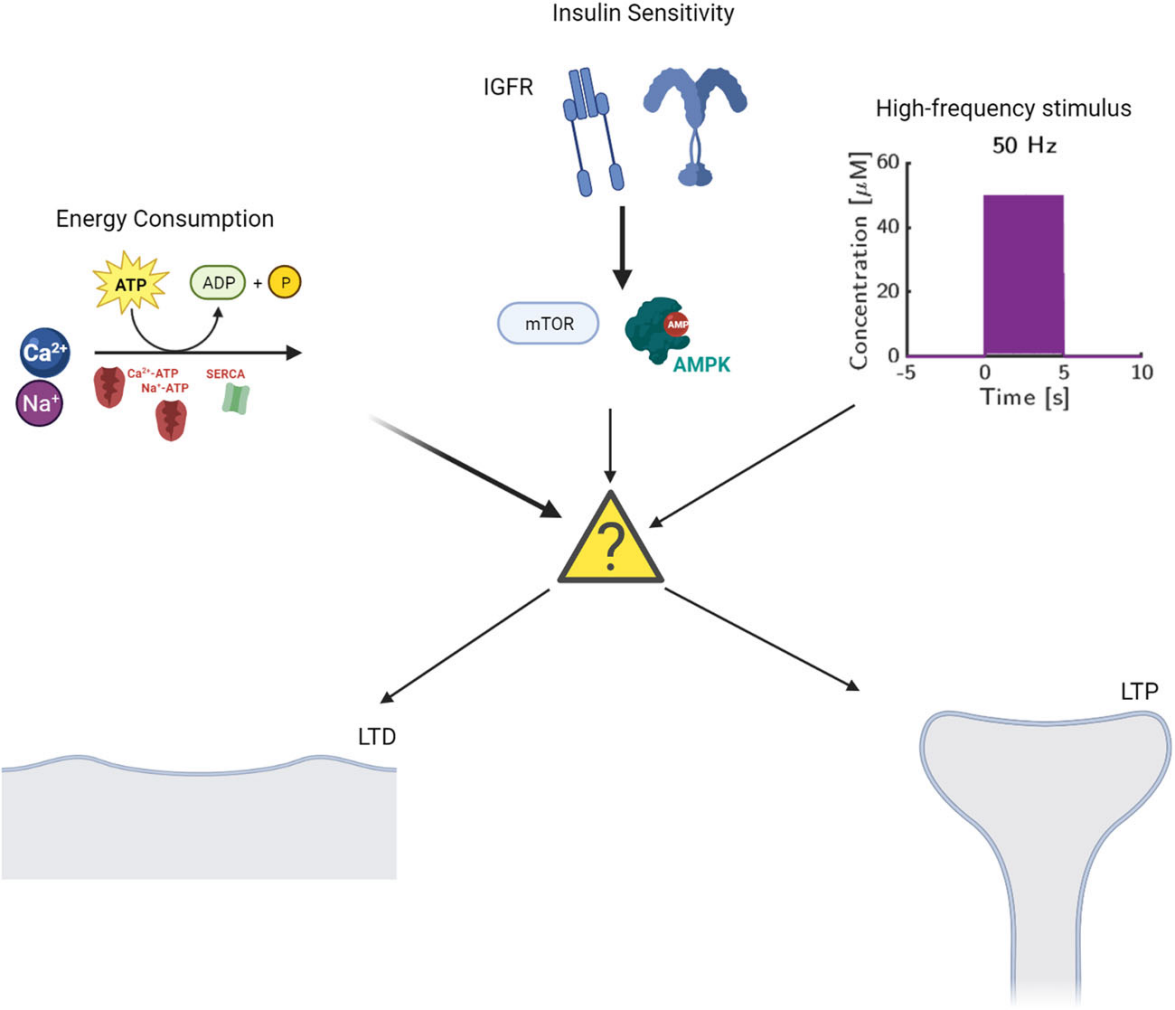
Cellular response to glutamate stimulus in neurons is regulated by insulin sensitivity and metabolics. A schematic for the various factors that lead into the decision between long-term potentiation and long-term depression.

We predict that the cellular response to energy consumption and external cellular signaling are frequency dependent and produce ranges of cellular stability behavior (Fig. 10). Specifically, the range of oscillations between low and high energy states can contribute to distinct signaling mechanisms. Furthermore, the oscillatory state may serve as a buffer range between random or noisy neuronal signals and high-frequency signals that invoke LTP in synapses. While it is known that AMPK hyperactivation can contribute to synaptic pruning^54^, these oscillations allow the system to reach higher rates of AMPK activation without raising the relative AUC. In this sense, oscillations controlled by the insulin system may regulate the levels of energetic stress that a neuron can withstand before triggering autophagy pathways. Although technically challenging, pharmacological experiments for AMPK and mTOR activators in dendritic spine could show a change in sensitivity to glutamatergic stimulus and subsequently affect LTP and LTD.

Models of synaptic plasticity can be generalized to phenomenological models (including rate-based models and spike timing-based models) and biophysical models which generally focus on calcium signaling and downstream signal transduction via CAMKII^7,55,56^. However, looking forward, models of synaptic plasticity can incorporate crosstalk effects of insulin on downstream components in biophysical models of synaptic plasticity. Insulin signaling has been shown to regulate neuroplasticity in both developing and adult brains^57^. While this can be explained by reports that insulin can transiently induce potentials via NMDA^58,59^, insulin has also been shown to impair NMDA-dependent LTP^60^. Similarly, insulin may also attenuate AMPAR signaling by inducing AMPA receptor internalization^61^. However, the mechanism of action for these disparate actions of insulin needs to be understood in the context of the dynamics of intracellular signaling molecules like AMPK and mTORC.

AMPK may be critical in the low-energy response of neurons, both from an intraceullar and systems level perspective^30,62^. While AMPK is critical for the integration of inputs, mTORC is linked to protein translation and autophagy, which can serve as outputs for influencing cellular state. mTORC1, while active in presynaptic neurons, is not expressed as heavily in postsynaptic dendritic spines^34^. mTORC2, is expressed in dendritic spines and activated during synaptic signaling^34,62,63^. There are additional reports that mTORC2 is essential for synaptic plasticity^34,64^. Due to the importance of insulin signaling and AMPK, further investigation of the kinetics of mTOR is vital for our understanding of cellular interactions leading to synaptic plasticity.

Future works in both modeling and experiments may focus more on building upon knowledge of synaptic plasticity. For modeling complex biochemical pathways, deterministic solver methods are feasible due to high molecule numbers^65^. However, incorporating stochastic solver methods may be able to capture biological behavior, which is particularly important in multi-scale problems like dendritic spines^3,66^ Finally, multivariate experimental measurements in spines during synaptic activity would be needed to constrain the models and test model predictions.

## Methods

### Model description

The model is constructed of 3 distinct modules (kinase activation, metabolism, and calcium signaling) with ATP consumption leading to AMPK activation as the key component that connects all three systems. Calcium dynamics are well studied in neuronal signaling literature, yet an often unexplored aspect is the crosstalk between metabolism and calcium signaling. A simple model representing glycolysis, oxidative phosphorylation, and energy consumption is used to provide a baseline for ATP consumption. Further, a model from skeletal muscle literature describing AMPK and mTOR activation downstream of insulin receptor activation is used to model the kinase activation. While there are definite differences in kinase expression and activation between neurons and skeletal muscle, both cells are excitable and undergo high frequencies of calcium stimulus. As more data specific to human neuron kinase activation becomes available, the parameterization of this aspect of the model may be improved, but in this work we focus on crosstalk applications between glutamatergic calcium signaling and kinase activation.

All differential equations are listed in Supplementary Table 1 and the fluxes for each reaction are listed in Supplementary Tables 2 to 4. Each parameter value used in the model is listed in Supplementary Tables 5 to 8. Here, we describe the various modules and the original sources for the reactions, where applicable. The system is composed of models representing AMPK and mTOR activation (Supplementary Table 2), calcium dynamics (Supplementary Table 3), and receptor dynamics NMDAR and AMPAR (Supplementary Table 4).

#### Calcium dynamics

Intracellular calcium is a critical cellular second messenger that is closely linked to the induction of synaptic plasticity^67^. In this model, we use reactions established in legacy literature models of neuronal calcium^1–3,43,68^. Calcium influx is initiated by glutamate binding to receptors on the PSD described in receptor models. Calcium release from the endoplasmic reticulum (ER) is triggered through binding of IP3 to IP3R on the ER membrane, as well as calcium-induced calcium release by ryanodine receptors (RyR)^69^. These species are modeled as concentrations in different cellular compartments and described in the equations listed below:

1 $$\frac{d[C{a}_{C}^{2+}]}{dt}={J}_{RYR}+{J}_{IP3}-{J}_{SERCA}+{J}_{PM}+{J}_{ER,Leak}-{J}_{Buff}$$

2 $$\frac{d[C{a}_{ER}^{2+}]}{dt}=-{J}_{RYR}-{J}_{IP3}+{J}_{SERCA}-{J}_{ER,Leak}-{J}_{ER,Buff}$$

Here, $C{a}_{C}^{2+}$ represents the calcium concentration in the cytosol and $C{a}_{ER}^{2+}$ represents the ER calcium concentration. Flux components of these differential equations are represented by Ryanodine (*J*_*RYR*_), IP3 Receptor (*J*_*IP*3_), SERCA pump (*J*_*SERCA*_), plasma membrane calcium-atpase (*J*_*PM*_), ER calcium leak (*J*_*ER*,*leak*_), and buffering terms for the cytosol (*J*_*Buff*_) and ER (*J*_*ER*,*Buff*_) and are fully described in Supplementary Table 3.

#### ATP production and consumption

ATP is rapidly consumed during synaptic activation due to the export of ions as well as many housekeeping reactions involved in synaptic plasticity^13,14,19,70^. ATP is generated by glycolysis and oxidative phosphorylation. Glycolysis is a well-studied pathway in which cells metabolize glucose into pyruvate to generate ATP^16^. Computational models of glycolytic activity in neurons have provided predictions for neurodegeneration, pathology, and development^71–73^. Pyruvate, the end product of glycolysis, is oxidized in mitochondria; in our model, oxidative phosphorylation is a function of mitochondrial potential computed by fluxes of the electron transport chain, as described by Beard^74^. In addition, as oxidative phosphorylation is enhanced by high cellular calcium concentrations, we have incorporated this dependence with a Hill-type equation on the flux which represents mitochondrial energy production, *J*_*OP*_. In contrast, ATP is hydrolyzed to ADP to provide energy for many reactions in the cytosol. In our model, both ATP production and consumption are modeled as mass action kinetics with kinetic parameters from the literature^74^. However, the rates of energy consumption are dependent on synaptic signaling^12,13^. In this model, we assume that the active synaptic energy consumption is correlated with the activity of active transporters, for example, SERCA and PMCA. In addition to active energy consumption from synapses, global energy consumption through maintenance and housekeeping reactions add up to the overall energy consumption rate in the model. ATP, ADP, and AMP are modeled in the cytosol as described in equations listed below:

3 $$\frac{d[AT{P}_{C}]}{dt}={J}_{Glyc}+{J}_{OP}-{J}_{AK}-{J}_{CK}-2{J}_{ATPCA}+{J}_{AMPK}$$

4 $$\frac{d[AD{P}_{C}]}{dt}=-{J}_{Glyc}-{J}_{OP}+2{J}_{AK}+{J}_{CK}+2{J}_{ATPCA}$$

5 $$\frac{d[AM{P}_{C}]}{dt}=-{J}_{AK}-{J}_{AMPK},$$

where ATP, ADP, and AMP are nucleotide concentrations over time. The fluxes modeled in these equations include: Glycolysis (*J*_*Glyc*_), Oxidative Phosphorylation (*J*_*OP*_), Adenylate Kinase (*J*_*AK*_), Creatine Kinase (*J*_*CK*_), ATP consumption by SERCA and PMCA pumps (*J*_*ATPCA*_), and AMPK activation(*J*_*AMPK*_) and are given in Supplementary Table 2.

#### Receptor models

Glutamate release into the synapse from the presynaptic cell is modeled as a series of step functions during the stimulus duration, with stimulus frequencies ranging from 0.1 to 100 Hz (Fig. 4a–c). The decay of glutamate within the synapse is based upon time constants from experiments by Clements^75^. NMDAR and AMPAR cascades are modeled as a multi-state receptor model with a prescribed voltage^76^. These equations are included in Supplementary Tables 5 to 8 and parameters in Supplementary Table 2.

#### AMPK and mTOR activation

AMPK and mTOR are coupled in an intricate feedback loop involving several other protein kinases. This system is directly downstream of insulin receptor signaling and is derived from Sadria^29^ (shown in Fig. 1). Sadria et al. developed an AMPK and mTOR signaling pathway for activity in cancer cells, parameterized from experimental data of mTOR activation in adipocytes of type 2 diabetes patients^77^. We have adapted the signaling network and differential equations for species including mTORC1, mTORC2, AKT, ULK1, SIRT1, IRS, and have integrated metabolic activation of AMPK by AMP/ATP ratio.

### Mathematical methods

The system of equations contains 60 species and 163 parameters. All equations were solved as a system of ordinary differential equations (ODE) using MATLAB’s built-in stiff solver, ode15s^78^. These equations were integrated with a maximum timestep of 0.1 s, relative integration tolerance of 10^−5^, using backward differentiation formulas with a maximum order of 4. Because each stimulus requires an instantaneous change in concentration and ODE solvers require a smooth, differentiable function, each stimulus was discretized to an individual ODE solution using the previous state as the subsequent initial condition, while glutamate concentration was increased to 0.1 mM. Before each pulse train simulation, the system was run to an approximate steady state, around 10,000 seconds with no glutamate stimulus. The results of this initial simulation are included in Supplementary Figure 1 and all model files are included in the public repository (https://github.com/aleung15/AMPKmTOR2022). In addition, we considered the role of noise in the glutamate stimulus of this system (Supplementary Figure 2). Noise in computational modeling of synaptic activity is frequently implemented due to the small molecule numbers and short timescales in synapses^2,3,42^. However, due to large variance in molecules in the system, highly nonlinear coupled system of equations, and the long timescales of interest, we focus instead on the deterministic response to a pulse train of glutamate stimulus.

### Sensitivity analysis

The dynamics of the model are dependent on both parameter values and initial conditions. The size and complexity of the system suggest nonlinear behavior such that changes in parameters for a single flux may lead to diverse response in the AMPK, AKT, mTORC1, and mTORC2 concentrations. To elucidate the role of each parameter in the system output we perform global sensitivity analysis. There are many methods of global sensitivity analysis, including correlation-based methods, variance-based methods, and derivative-based method^79^. In this work, we use a Latin Hypercube Sampling method to determine a sampling plan across a parameter range of 20% of the original parameter values^80^.

Each parameter set is then simulated to an apparent steady state and values for AMPK, AKT, mTORC1, and mTORC2 are compared to the default values. We then perform a partial correlation analysis to determine the Partial Rank Correlation Coefficient (PRCC), which represents the linear dependence between the parameters and output variables. This approach is suitable for the analysis of the steady-state simulation, since many of the non-linearities are introduced through the rapid glutamatergic and calcium signaling^81^.

### Determination of system metrics

We use the following metrics to compare model outputs for different inputs: steady state, time to steady state, maximum amplitude, and area under the curve. Steady state was determined by taking the derivative of phosphorylated AMPK, mTORC1, and mTORC2 with respect to time. After the mean magnitude of the derivative was computed to be lower than 1 × 10^−6 ^μM/s for a period of 30 s, the species was determined to be at steady state. For the time to steady state, the same computation was done, however the time at which the derivative was lower than 1 × 10^−6 ^μM/s was determined to be the time to steady state. Maximum amplitude was computed to be the percent change relative to the steady-state value for each condition. Finally, area under the curve (AUC) was numerically determined by the integrating the predicted phosphorylation time-course over a period of 200 s. In heatmaps Figs. 7 to 9, the values of the AUC’s are taken relative to a system without any stimulus or changes in parameters, then normalized to the maximum AUC computed. This was done in order to reduce the range of values on the color bar to be more reflective of the changes in simulation conditions rather than the steady-state concentrations of the species.

### Reporting summary

Further information on research design is available in the Nature Research Reporting Summary linked to this article.

## Supplementary information

Supplementary Materials

Reporting Summary

## Data Availability

All data needed to reproduce this work are included in public repository hosted at: https://github.com/aleung15/AMPKmTOR2022.

## Code availability

All code and data needed to reproduce this work are included in public repository hosted at: https://github.com/aleung15/AMPKmTOR2022.
