# Supplementary material for: Computational modeling of AMPK and mTOR crosstalk in glutamatergic synapse calcium signaling
Source: NPJ Syst Biol Appl. 2023 Jul 17;9:34. doi: 10.1038/s41540-023-00295-4 (PMC10352260; doi:10.1038/s41540-023-00295-4)
Supplement: Supplementary file 1 — Supplementary Materials [file 41540_2023_295_MOESM1_ESM.pdf]

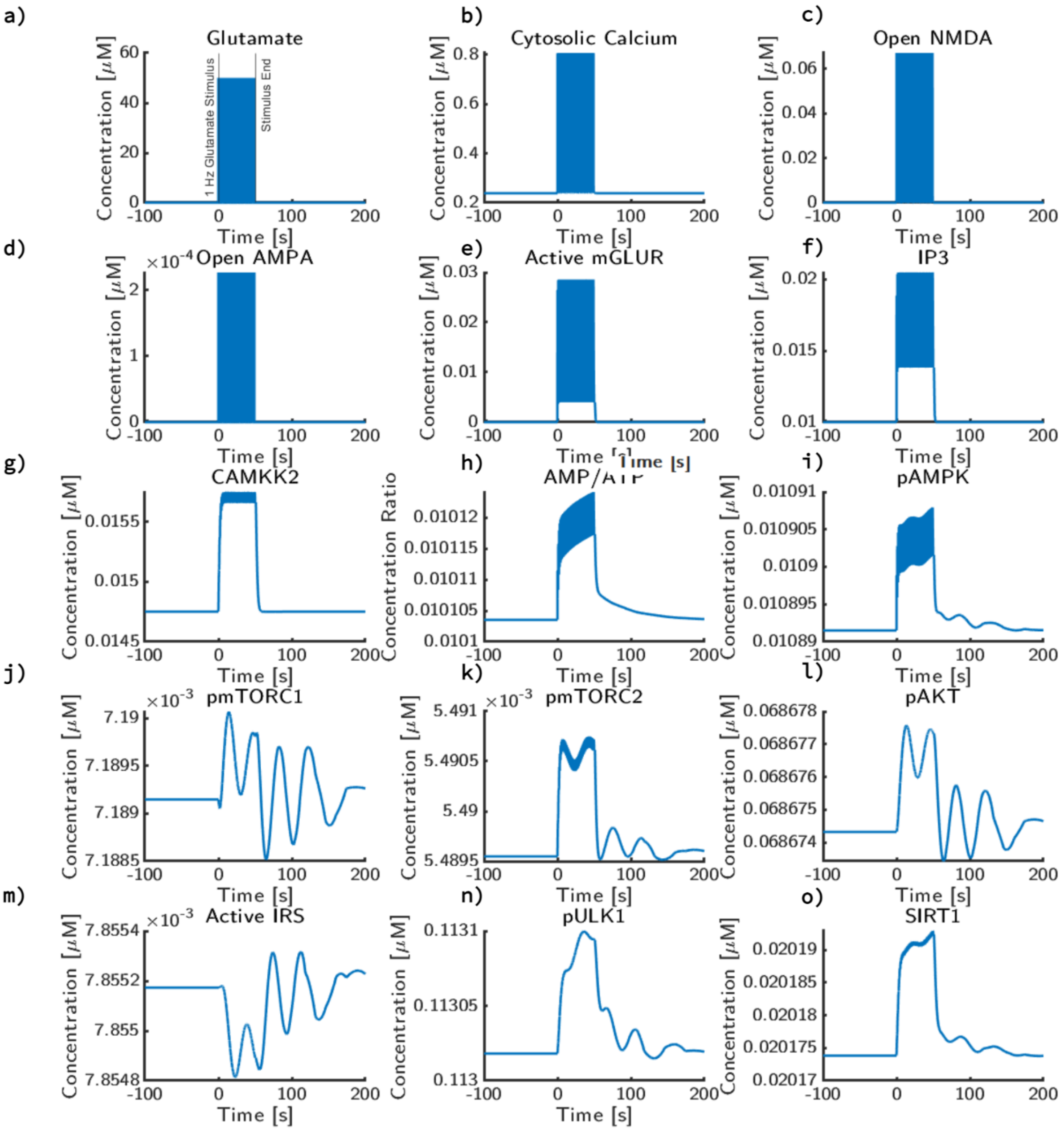

Supplementary Figure 1: **Equilibration simulation for system without glutamatergic stimulus.** The system recieved a single pulse of glutamate and was allowed to equilibrate for 2000 seconds. Trajectories are plotted for:**a)** glutamate, **b)** cytosolic calcium, **c)** open NMDA Receptors, **d)** open AMPA receptors, **e)** active mGLUR receptor, **f)** IP3, **g)** CAMKK2, **h)** AMP/ATP ratio, **i)** phosphorylated AMPK, **j)** phosphorylated mTORC1, **k)** phosphorylated mTORC2, **l)** phosphorylated AKT, **m)** active IRS, **n)** phosphorylated ULK1, **o)** SIRT1.

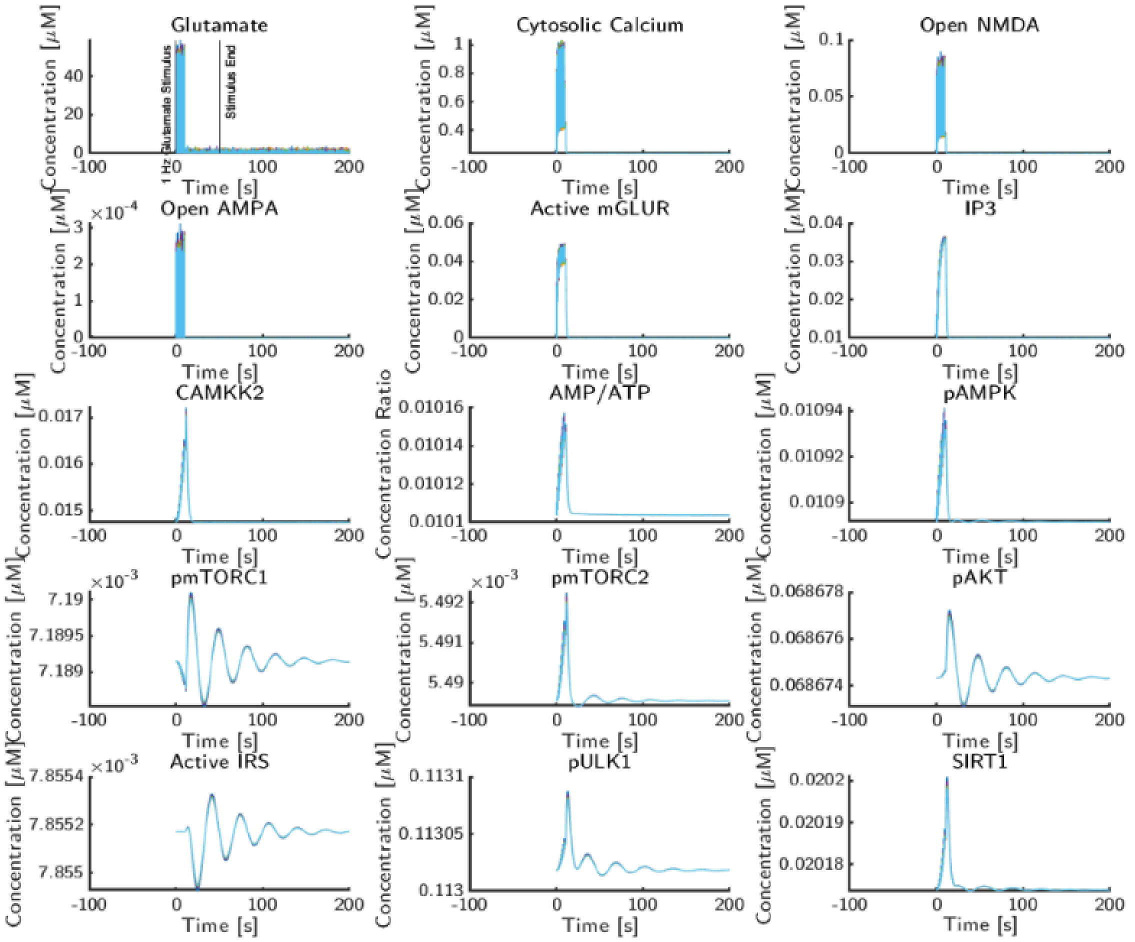

Supplementary Figure 2: **Stochastic simulation for system with noise applied to glutamatergic stimulus.** The system received a 1 hz pulse train of stimulus similar to those depicted in Supplementary Figure 3. However, a gaussian white noise was also applied to glutamate stimulus, leading to variance in system response. 20 trajectories are plotted for:**a)** glutamate, **b)** cytosolic calcium, **c)** open NMDA Receptors, **d)** open AMPA receptors, **e)** active mGLUR receptor, **f)** IP3, **g)** CAMKK2, **h)** AMP/ATP ratio, **i)** phosphorylated AMPK, **j)** phosphorylated mTORC1, **k)** phosphorylated mTORC2, **l)** phosphorylated AKT, **m)** active IRS, **n)** phosphorylated ULK1, **o)** SIRT1.

Equations and Tables

Supplementary Table 1: Differential Equations used in the model

Index	Equation
	Nucleotides and AMPK
1	$\frac{d}{dt}[ATP] = -r1 + r2 - AK - CK - 2 * J_{ATP,Ca} + rc,$
2	$\frac{d}{dt}[ADP] = r1 - r2 + 2AK + CK + 2J_{ATP,Ca},$
3	$\frac{d}{dt}[AMP] = -AK - r20 - rc,$
4	$\frac{d}{dt}[PCr] = CK,$
5	$\frac{d}{dt}[Pi] = r1 - r2,$
6	$\frac{d}{dt}[AMPK] = -rc + JM_{13} + JM_{14} - JM_{15},$
7	$\frac{d}{dt}[pAMPK] = rc + JM_{15} - JM_{13} - JM_{14},$
	Calcium and IP3
8	$\frac{d}{dt}[Glut] = -200 * Glut,$
9	$\frac{d}{dt}[Ca_{ER}] = (J_{SERCA} - J_{IP3R} - J_{RYR} - J_{ER,leak}) - J_{ER,Buff},$
10	$\frac{d}{dt}[Ca_{C}] = -(J_{SERCA} - J_{IP3R} - J_{RYR} + J_{ER,leak}) + J_{PM} - J_{BuffCa} - J_{CaMBind},$
11	$\frac{d}{dt}[w] = J_{w},$
12	$\frac{d}{dt}[Ri] = J_{Ri},$
13	$\frac{d}{dt}[R2] = J_{R2},$
14	$\frac{d}{dt}[DIM] = J_{DIM},$
15	$\frac{d}{dt}[DAG] = J_{DAG},$
16	$\frac{d}{dt}[DIMp] = J_{DIMp},$
17	$\frac{d}{dt}[PKC] = J_{PKC},$
18	$\frac{d}{dt}[IP3] = J_{IP} - k_{deg}(IP3 - IP3_{0}),$
	Buffer and Calmodulin
19	$\frac{d}{dt}[B] = -J_{BuffCa}$
20	$\frac{d}{dt}[BCa] = +J_{BuffCa}$
21	$\frac{d}{dt}[BuffER] = J_{ER,Buff}$
22	$\frac{d}{dt}[CaM] = -J_{CaMBind} + J_{CaMDisc}1$
23	$\frac{d}{dt}[CaCaM] = J_{CaMBind} - J_{CaMDisc}$
24	$\frac{d}{dt}[CaMKK2] = -J_{CAMKK2,Act} + J_{CAMKK2,Deac}$
25	$\frac{d}{dt}[CaMKK2_{act}] = J_{CAMKK2,Act} - J_{CAMKK2,Deac}$
	Insulin System
26	$\frac{d}{dt}pIR] = JM2 - JM1$

27	$\frac{d}{dt}[pIR] = JM1 - JM2$
28	$\frac{d}{dt}[IRS] = JM4 + JM16 - JM3 - JM15$
29	$\frac{d}{dt}[pIRS] = JM3 - JM4$
30	$\frac{d}{dt}[iIRS] = JM15 - JM16$
31	$\frac{d}{dt}[AKT] = JM6 - JM5$
32	$\frac{d}{dt}[pAKT] = JM5 - JM6$
33	$\frac{d}{dt}[mTORC1] = JM8 - JM7 - JM13$
34	$\frac{d}{dt}[pmTORC1] = JM7 - JM8$
35	$\frac{d}{dt}[mTORC2] = JM10 - JM9 - JM14$
36	$\frac{d}{dt}[pmTORC2] = JM9 - JM10$
37	$\frac{d}{dt}[mTORC1 - DEPTOR] = JM13$
38	$\frac{d}{dt}[mTORC2 - DEPTO] = JM14$
39	$\frac{d}{dt}[DEPTOR] = JM12 - JM11 - JM13 - JM14$
40	$\frac{d}{dt}[pDEPTOR] = JM11 - JM12$
41	$\frac{d}{dt}[SIRT1] = JM19$
42	$\frac{d}{dt}[ULK1] = JM21 - JM20$
43	$\frac{d}{dt}[pULK1] = JM20 - JM21$
	AMPA and NMDA
44	$\frac{d}{dt}[NMDA_{C0}] = -JN1,$
45	$\frac{d}{dt}[NMDA_{C1}] = +JN1 - JN2,$
46	$\frac{d}{dt}[NMDA_{C2}] = +JN2 - JN3 - JN4,$
47	$\frac{d}{dt}[NMDA_{D}] = JN3,$
48	$\frac{d}{dt}[NMDA_{O}] = JN4,$
49	$\frac{d}{dt}[AMPA_{U}] = -JA1,$
50	$\frac{d}{dt}[AMPA_{M}] = +JA1 - JA2 - JA4,$
51	$\frac{d}{dt}[AMPA_{C}] = JA2 - JA3 - JA5,$
52	$\frac{d}{dt}[AMPA_{O}] = JA3 - JA6,$
53	$\frac{d}{dt}[AMPA_{D1}] = JA4 - JA7,$
54	$\frac{d}{dt}[AMPA_{D2}] = JA5 + JA7 - JA8,$
55	$\frac{d}{dt}[AMPA_{D3}] = JA6 + JA8,$
56	$[V] = -65 + BPAP + EPSP + AMPA_{E}PSP,$

Supplementary Table 2: AMPK, mTOR, and Metabolism Reactions

Rx	Reaction	Flux Equation	Note	Ref
r1	$ATP \rightarrow ADP$	$k_{HYD}ATP$	Hydrolysis	[82]
r2	$ADP \rightarrow ATP$	$\frac{VmaxOP*(ADP/KADP)^n H}{1+(ADP/KADP)^{nH}}$	Oxidative Phosphorylation	[82]
r3	$ADP + PCr \rightarrow ATP + Cr$	$\frac{VforCK*ADP*PCr/(Kia*Kb)}{1+\frac{ADP}{Kia}+\frac{PCr}{Kib}+\frac{ATP}{Kiq}+(ADP*\frac{PCr}{Kia*Kb})+((TCr-PCr)*\frac{ATP}{Kiq*Kp})}$	Creatine Kinase	[82]
r4	$ATP + Cr \rightarrow ADP + PCr$	$\frac{vCKr*ATP*(TCr-PCr)/(Kiq*Kp)}{1+\frac{ADP/Kia}{+}\frac{(PCr}{Kib}+\frac{ATP}{Kiq})+(ADP\frac{PCr}{(Kia*Kb})+((TCr-PCr)*\frac{ATP}{Kiq*Kp})}$	Creatine Kinase, Reverse	[82]
r5f	$ADP + ADP \rightarrow ATP + AMP$	$\frac{vAK*ATP*(AMP)/(kmt*kmm)}{1+(ATP/kmt)+(AMP/kmm)+(ATP*AMP/(kmt*kmm))+(2*ADP/kmd)+(ADP^2/kmd^2)}$	Adenylate Kinase	[82]
r5r	$ATP + AMP \rightarrow ADP + ADP$	$\frac{VrevAK*ADP^2/kmd^2}{1+(ATP/kmt)+(AMP/kmm)+(ATP*AMP/(kmt*kmm))+(2*ADP/kmd)+(ADP^2/kmd^2)}$	Adenylate Kinase	[82]
r6	$AMP + AMPK \rightarrow ATP + pAMPK$	$k_{fAMPK}AMPAMPK - k_{rAMPK}ATPpAMPK$	AMPK act. Reaction	[82]
r7	$AKT \rightarrow pAKT$	$\frac{V_{pAKT}*pAKT}{K_{m,pAKT}+pAKT}$	AKT Basal act.	[29]
r8	$pAKT \rightarrow AKT$	$\frac{K_{AKTpmTOR}*pmTORC2*AKT}{K_{m,AKT-pmTOR}+AKT}$	AKT Deact. by mTORC2	[29]
r9	$pmTORC1 \rightarrow mTORC1$	$\frac{K_{pmTORC1}+K_{pmTORC1AMPK}*pAMPK*pmTORC1}{K_{mpmTORC1A}+pmTORC1}$	mTORC1 deact. by AMPK	[29]
r10	$pmTORC1 \rightarrow mTORC1$	$\frac{K_{pmTORC1ULK1}*pULK1*pmTORC1}{K_{m,pmTORULK}+pmTORC1}$	mTORC1 deact. by ULK1	[29]
r11	$pmTORC1 \rightarrow mTORC1$	$\frac{K_{MAKT}*pAKT*mTORC1}{K_{m,MAKT}+mTORC1}$	mTORC1 act. by AKT	[29]
r12	$mTORC1 + DEP \rightarrow mTORC1 - DEP$	$K_{fDEP1} * mTORC1 * DEP - KrDEP1 * mTORC1 - DEP$	mTORC1 binding by DEP	[29]
r13	$pmTORC2 \rightarrow mTORC2$	$\frac{V_{mTORC2}*pmTORC2}{Km_{pmTORC2}+pmTORC2}$	mTOR basal deact.	[29]
r14	$mTORC2 \rightarrow pmTORC2$	$\frac{k_{M2AMPK}*pAMPK*mTORC2}{K_{m,m2AMPK}+mTORC2}$	mTORC2 act. by AMPK	[29]
r15	$mTORC2 + DEP \rightarrow mTORC2 - DEP$	$K_{f,DEP2} * mTORC2 * DEP - KrDEP2 * mTORC2 - DEP$	mTORC2 binding by DEP	[29]
r16	$pDEP \rightarrow DEP$	$\frac{V_{pDEP}*pDEP}{K_{m,pDEP}+pDEP}$	DEP dephos.	[29]
r17	$DEP \rightarrow pDEP$	$\frac{K_{DEPM1}*pmTORC1*DEP}{K_{m,DEPM1}+DEP}$	DEP act. with mTORC1	[29]
r18	$DEP \rightarrow pDEP$	$\frac{K_{DEPM2}*pmTORC2*DEP}{K_{m,DEPM2}+DEP}$	DEP act. with mTORC2	[29]
r19	$pAMPK \rightarrow AMPK$	$\frac{K_{pAMPK}+K_{pAMPKULK}*pULK1*pAMPK}{K_{m,pAMPK}+pAMPK}$	AMPK deact. by ULK1	[29]
r20	$pAMPK \rightarrow AMPK$	$\frac{K_{pAMPKM1}*pmTORC1*pAMPK}{K_{m,pAMPK}+pAMPK}$	AMPK deact. by mTORC1	[29]
r21	$AMPK \rightarrow pAMPK$	$\frac{K_{AMPK}+KAMPKSIRT*SIRT1*AMPK}{Km,AMPK+AMPK}$	AMPK act. by SIRT1	[29]
r22	$\rightarrow SIRT$	$\frac{K_{SIRT1}+KSIRT1_A*pAMPK*SIRT1_T-SIRT1}{K_{m,SIRT1}+SIRT1_T-SIRT1}$	SIRT act. by AMPK	[29]
r23	$ULK1 \rightarrow pULK1$	$\frac{K_{pULK1}+KpULK1m1*pmTORC1*pULK1}{K_{mpULK1}+pULK1}$	ULK act. by mTORC1	[29]
r24	$pULK1 \rightarrow ULK1$	$\frac{K_{ULK1}+KULK1AMPK*pAMPK*ULK1}{K_{mULK1}+ULK1}$	ULK deact. by AMPK	[29]
r25	$AKT \rightarrow pAKT$	$\frac{IRS*v_{akti}*AKT}{k_{m-akt-i}+AKT}$	Basal IRS act. of AKT	[29]
r26	$mTORC2 \rightarrow pmTORC2$	$\frac{IRS*v_{mtori}*mTORC2}{k_{m-mtor-i}+mTORC2}$	Basal IRS act. mTORC2	[29]

Supplementary Table 3: Calcium Submodel Reactions

Reaction No.	Reaction	Flux	Parameters	Citation	Note
J_{SERCA}	$Ca_c^{2+} + ATP_e \rightarrow Ca_e^{2+}$	$J_{SERCA} = V_{SERCA} \frac{Ca_c^2}{K_p^2 + Ca_c^2} \frac{ATP_e}{K_{ds} + ATP_e}$		[83]	
J_{IP3R}	$Ca_e^{2+} \rightarrow Ca_c^{2+}$	$J_{IP_3R} = (k_{IP}b + k_{IP}IPR_{act})(Ca_e^{2+} - Ca_c^{2+})$	$K_f = 10, \gamma = 10$	[83]	IPR (Table 3)
J_{NMDA}	$\phi \rightarrow Ca_c^{2+}$	$J_{NMDA} = g_{NMDA} NMDA_O \frac{1}{1+(e^{0.092V})*0.28}$		[76]	NMDA
J_{RYR}	$\phi \rightarrow Ca_c^{2+}$	$J_{RYR} = (V_{RYR} P_O + V_{leak})(Ca_e^{2+} - Ca_c^{2+})$		[84]	Ryanodine
J_{PMCA}	$Ca_c^{2+} + ATP_e \rightarrow \phi$	$J_{PMCA} = V_{PMCA} \frac{Ca_c^2}{K_p^2 + Ca_c^2}$		[85]	PMCA
J_{Buff}	$B + Ca_c^{2+} \rightarrow BCa_c^{2+}$	$J_{Buff} = k_{Buff}(Ca_c B - k_{eq,Buff} BCa)$		[86]	Buffering

Supplementary Table 4: PSD Receptor Submodel Reactions

Flux	Refs
NMDA	
$J_{N1} = k_{1f}[Glut][U] - k_{1r}[M]$	[76]
$J_{N2} = k_{2f}[Glut][M] - k_{2r}[C3]$	[76]
$J_{N3} = k_{3f}[C3] - k_{3r}[C2]$	[76]
$J_{N4} = k_{4f}[C2] - k_{4r}[C1]$	[76]
$J_{N5} = k_{5f}[C1] - k_{5r}[O]$	[76]
$J_{N6} = k_{6f}[C3] - k_{6r}[D1]$	[76]
$J_{N7} = k_{7f}[C2] - k_{7r}[D2]$	[76]
$J_{N8} = k_{8f}[C1] - k_{8r}[O]$	[76]
mGluR	
$J_{LR} = -k_b R_2 G^2 + k_u DIM$	[68]
$J_{DIM} = V_P \frac{DIM_p}{K_{mP} + DIM_p} - V_{PKC} \frac{PKC \cdot DIM}{K_{mPKC} + DIM}$	[68]
$J_{DIM_p} = \frac{R_{tot} - \sqrt{K_{dim} R_2} - 2R_2 - 2DIM}{2}$	[68]
$J_{DAG} = \frac{dPKC}{dt} = k_{aPKC} \frac{DAG}{K_{mDAG} + DAG}(1 - PKC) - k_{dPKC} PKC$	[68]
$J_{IP_3} = k_{PLC_I} DIM$	[68]
$J_{A1} = k_{A1f}[Glut][U] - k_{A1r}[M]$	[76]
$J_{A2} = k_{A2f}[Glut][M] - k_{A2r}[C]$	[76]
$J_{A3} = k_{A3f}[C] - k_{A3r}[O]$	[76]
$J_{A4} = k_{A4f}[M] - k_{A4r}[D1]$	[76]
$J_{A5} = k_{A5f}[C] - k_{A5r}[D2]$	[76]
$J_{A6} = k_{A6f}[O] - k_{A6r}[D]$	[76]
$J_{A7} = k_{A7f}[D1][Glut] - k_{A7r}[D2]$	[76]
$J_{A8} = k_{A8f}[D2] - k_{A8r}[D3]$	[76]

Supplementary Table 5: Parameter Table

#	Parameter	Value	Ref	#	Parameters	Value	Ref
	Calcium Module						
31	$k1$	30	[83]	32	b	0.001	[83] [Altered]
33	Ki	5 [µM]	[84]	34	Ka	3 [µM]	[84]
35	IRa	EQN	[83]	36	k_{-}	0.02 [1/s]	[83]
37	k_{-}	20 [1/µM^{4}s]	[84]	38	V_{DAG}	0.0325 [1/µM]	[84]
39	kb	0.1 [1/µM^{2}s]	[68]	40	ku	2 [1/s]	[68]
41	Vp	0.05 [µM/s]	[68]	42	Kmp	5×10^{-4} [µM]	[68]
43	$Vpkc$	0.2 [µM/s]	[68]	44	$Kmpkc$	5×10^{-4} [µM]	[68]
45	$kapkc$	0.2 [1/s]	[68]	46	$kdpkc$	20 [1/s]	[68]
47	$kplcI$	3 [1/s]	[68]	48	$kplcD$	3 [1/s]	[68]
49	$KmDAG$	6 [µM]	[68]	50	$VRYR$	0.0050 [1/s]	[84]
51	Kar	0.0192 [1/s]	[84]	52	Kbr	0.2573 [1/s]	[84]
53	Kcr	0.0571 [1/s]	[84]	54	Kdr	0.1 [1/s]	[84]
55	V_{SERCA}	120 [µM/s]	[83]	56	$KpSERCA$	6 [µM]	[**Wacquier2016**]
74	Km_{ext}	0.1 [µM^2]	[85]	75	k_{deg}	5 [1/s]	[83]
76	$kpmleak$	0.5 [1/s]	[85]	77	k_{bcaf}	0.1 [1/(µM s)]	[1]
78	k_{bcar}	1 [1/s]	[1]	79	k_{ERBf}	50 [1/(µM s)]	[86]
80	k_{ERBr}	3 [1/s]	[86]	81	ker_{leak}	0.1 [1/s]	[To Sustain]
99	k_{cam_f}	0.001 [1/(µM s)]	[86]	100	k_{cam_r}	1 [1/s]	[86]
101	Vm_{kk2}	0.01 [µM/s]	[87]	102	Km_{kk2}	0.01 [µM]	[87]
103	Ka_{kk2}	0.005 [µM]	[87]	104	$kf_{CaMDisc}$	1 [1/s]	[86]

Supplementary Table 6: Parameter Table

#	Parameter	Value	Ref	#	Parameters	Value	Ref
	PSD Module						
57	Rb	5 1/(µM s)]	[76]	58	Ru	46.5 $[1/s]$	[76]
59	Rd	8.4 $[1/s]$	[76]	60	Rr	6.8 $[1/s]$	[76]
61	Ro	46.5 $[1/s]$	[76]	62	Rc	73.8 $[1/s]$	[76]
63	τ_{ess}	0.05 $[s]$	[7]	64	τ_{esf}	0.005 $[s]$	[7]
65	τ_{bss}	0.025 $[s]$	[7]	66	τ_{bsf}	0.003 $[s]$	[7]
67	$\tau_{delay,bp}$	0.002 $[s]$	[7]	68	s	10 $[mV]$	[7]
69	V_{rev}	-65 $[mV]$	[3]	70	N_{NMDA}	1	[76]
71	$BPAP_{max}$	40 $[mV]$	[7]	72	G_{NMDA}	-65.6×10^{-6} $[nS]$	[7]
73	k_{ext}	1.3×10^{-6} $[1/s]$	[Altered] [85]	82	G_{AMPA}	15 $[nS]$	[7]
83	$kAMPA_{1f}$	1.8 [1/(µM s)]	[76]	84	$kAMPA_{2f}$	10 [1/(µM s)]	[76]
85	$kAMPA_{3f}$	1.6×10^{-4} $[1/s]$	[76]	86	$kAMPA_{4f}$	7×10^{2} $[1/s]$	[76]
87	$kAMPA_{5f}$	1×10^{2} $[1/s]$	[76]	88	$kAMPA_{6f}$	3×10^{2} $[1/s]$	[76]
89	$kAMPA_{7f}$	10 $[1/s]$	[76]	90	$kAMPA_{8f}$	1.6×10^{4} $[1/s]$	[76]
91	$kAMPA_{1r}$	2.4×10^{3} $[1/s]$	[76]	92	$kAMPA_{2r}$	1×10^{4} $[1/s]$	[76]
93	$kAMPA_{3r}$	5×10^{3} $[1/s]$	[76]	94	$kAMPA_{4r}$	1.5×10^{2} $[1/s]$	[76]
95	$kAMPA_{5r}$	2.1 $[1/s]$	[76]	96	$kAMPA_{6r}$	15 $[1/s]$	[76]
97	$kAMPA_{7r}$	1×10^{3} $[1/s]$	[76]	98	$kAMPA_{8r}$	1.2×10^{4} $[1/s]$	[76]

Supplementary Table 7: Parameter Table

#	Parameter	Value	Ref	#	Parameters	Value	Ref
	ATP Energetics						
1	K_{ADP}	500 [µM]	[82]	2	$VmaxOP$	0.5 $[mM/s]$	[82]
3	nH	2.568	[82]	4	k_{HYD}	2.6×10^{-2} $[mM/s]$	[82]
7	vAK	14.66 $[mM/s]$	[82]	8	kmt	0.27 $[mM]$	[82]
9	kmd	0.35 $[mM]$	[82]	10	kmm	0.32 $[mM]$	[82]
11	$keqadk$	0.744	[82]	12	$vmax20$	2×10^{-2} $[mM/s]$	[82]
13	$vmax21$	1×10^{-4}	[82]	14	$km20$	1.4 $[mM]$	[82]
15	$km21$	6.7×10^{-2} $[mM]$	[82]	16	$k12f$	1 $[1/(mMs)]$	[82]
17	$k12r$	1.82×10^{-2} $[1/s]$	[82]	18	$k13f$	1 $[1/(mMs)]$	[82]
19	$k13r$	1.82×10^{-2} $[1/s]$	[82]	20	$kaicar$	4×10^{-4} $[1/s]$	[82]
21	$kAKT$	3×10^{-3} $[1/s]$	[82]	22	$kcatAMPK$	34.56 $[1/s]$	[82]
23	V_{CK}	1×10^{2}	[82]	24	Kb	1.11 $[mM]$	[82]
25	Kia	0.135 $[mM]$	[82]	26	Kib	3.9 $[mM]$	[82]
27	Kiq	3.5 $[mM]$	[82]	28	$Kp(ATP)$	3.8 $[mM]$	[82]
29	$KeqCK$	1.77×10^{2}	[82]	30	TCr	39 $[mM]$	[82]

Supplementary Table 8: Parameter Table

#	Parameter	Value	Ref	#	Parameters	Value	Ref
	mTOR Module						
105	V_{IR}	9.67 $[nM/s]$	[29]	106	Km_{IR}	94.9 $[nM/s]$	[29]
107	V_{pIR}	26.2 $[nM/s]$	[29]	108	Km_{pIR}	35.0 $[nM]$	[29]
109	K_{IRSpIR}	3.57 $[1/s]$	[29]	110	Km_{IRSpIR}	50.1 $[nM]$	[29]
111	V_{pIRS}	18.4 $[1/s]$	[29]	112	Km_{pIRS}	50.0 $[nM]$	[29]
113	$K_{AKT,IRS}$	1.243 $[1/s]$	[29]	114	$Km_{AKT,IRS}$	6.27 $[nM]$	[29]
115	$K_{AKT,pM2}$	0.564 $[1/s]$	[29]	116	$Km_{AKT,pM2}$	5.23 $[nM]$	[29]
117	V_{pAKT}	16.2 $[nM/s]$	[29]	118	Km_{pAKT}	33.6 $[nM]$	[29]
119	$Km_{pM1,pAKT}$	0.594 $[1/s]$	[29]	120	$K_{pM1,pAKT}$	2.32 $[1/s]$	[29]
121	K_{pm1}	50.2 $[1/s]$	[29]	122	$K_{pM1,pAMPK}$	0.0238 $[1/s]$	[29]
123	$Km_{pM1,pAMPK}$	2.14 $[nM]$	[29]	124	$K_{pM1,ULK}$	0.0113 $[1/s]$	[29]
125	$Km_{pM1,ULK}$	1.99 $[nM]$	[29]	126	$K_{M2,IRS}$	13.3 $[1/s]$	[29]
127	$Km_{M2,IRS}$	160 $[nM]$	[29]	128	$K_{M2,pAMPK}$	1.57 $[1/s]$	[29]
129	$Km_{M2,pAMPK}$	160 $[nM]$	[29]	130	$V_{pmTORC2}$	25.4 $[nM/s]$	[29]
131	$Km_{pmTORC2}$	8.20 $[nM]$	[29]	132	K_{DEPM1}	0.995 $[1/s]$	[29]
133	Km_{DEPM1}	120.1 $[nM]$	[29]	134	K_{DEPM2}	0.279 $[1/s]$	[29]
135	Km_{DEPM2}	10.6 $[nM]$	[29]	136	$V_{pDEPTOR}$	54.1 $[nM/s]$	[29]
137	$Km_{pDEPTOR}$	8.23 $[nM]$	[29]	138	K_{M1DEPf}	0.0163 $[1/s]$	[29]
139	K_{M1DEPd}	0.120 $[1/s]$	[29]	140	K_{M2DEPf}	0.144 $[1/s1/s]$	[29]
141	K_{M2DEPd}	0.120 $[1/s]$	[29]	142	$K_{IRS,iIRS}$	1.84 $[1/s]$	[29]
143	$Km_{IRS,iIRS}$	50.1 $[nM]$	[29]	144	V_{iIRS}	13.5 $[nM/s]$	[29]
145	Km_{iIRS}	50.1 $[nM]$	[29]	146	K_{AMPK}	100 $[1/s]$	[29]
147	$K_{AMPK,SIRT1}$	84.3 $[1/s]$	[29]	148	Km_{AMPK}	124 $[nM]$	[29]
149	K_{pAMPK}	22.4 $[1/s]$	[29]	150	$K_{pAMPK,pULK1}$	473 $[1/s]$	[29]
151	$K_{pAMPK,pmTORC1}$	18.9 $[1/s]$	[29]	152	Km_{pAMPK}	100 $[nM]$	[29]
153	K_{SIRT1}	2.32 $[1/s]$	[29]	154	$K_{SIRT1,pAMPK}$	0.727 $[1/s]$	[29]
155	Km_{SIRT1}	3.77 $[nM]$	[29]	156	$K_{SIRT1,r}$	0.502 $[1/s]$	[29]
157	K_{ULK1}	39.2 $[1/s]$	[29]	158	$K_{ULK1,pAMPK}$	2.05 $[1/s]$	[29]
159	Km_{ULK1}	98.9 $[nM]$	[29]	160	K_{pULK1}	0.131 $[1/s]$	[29]
161	$K_{pULK1,pmTORC1}$	6.00 $[1/s]$	[29]	162	Km_{pULK1}	23.5 $[nM]$	[29]
